# Meiotic Cohesin SMC1β Provides Prophase I Centromeric Cohesion and Is Required for Multiple Synapsis-Associated Functions

**DOI:** 10.1371/journal.pgen.1003985

**Published:** 2013-12-26

**Authors:** Uddipta Biswas, Cornelia Wetzker, Julian Lange, Eleni G. Christodoulou, Michael Seifert, Andreas Beyer, Rolf Jessberger

**Affiliations:** 1 Institute of Physiological Chemistry, Medical Faculty Carl Gustav Carus, Technische Universität Dresden, Dresden, Germany; 2 Molecular Biology Program, Memorial Sloan-Kettering Cancer Center, New York, New York, United States of America; 3 Biotechnology Center, TU Dresden, Dresden, Germany; 4 Center for Regenerative Therapies Dresden, Dresden, Germany; Stowers Institute for Medical Research, United States of America

## Abstract

Cohesin subunit SMC1β is specific and essential for meiosis. Previous studies showed functions of SMC1β in determining the axis-loop structure of synaptonemal complexes (SCs), in providing sister chromatid cohesion (SCC) in metaphase I and thereafter, in protecting telomere structure, and in synapsis. However, several central questions remained unanswered and concern roles of SMC1β in SCC and synapsis and processes related to these two processes. Here we show that SMC1β substantially supports prophase I SCC at centromeres but not along chromosome arms. Arm cohesion and some of centromeric cohesion in prophase I are provided by non-phosphorylated SMC1α. Besides supporting synapsis of autosomes, SMC1β is also required for synapsis and silencing of sex chromosomes. In absence of SMC1β, the silencing factor γH2AX remains associated with asynapsed autosomes and fails to localize to sex chromosomes. Microarray expression studies revealed up-regulated sex chromosome genes and many down-regulated autosomal genes. SMC1β is further required for non-homologous chromosome associations observed in absence of SPO11 and thus of programmed double-strand breaks. These breaks are properly generated in *Smc1β^−/−^* spermatocytes, but their repair is delayed on asynapsed chromosomes. SMC1α alone cannot support non-homologous associations. Together with previous knowledge, three main functions of SMC1β have emerged, which have multiple consequences for spermatocyte biology: generation of the loop-axis architecture of SCs, homologous and non-homologous synapsis, and SCC starting in early prophase I.

## Author Summary

The generation of mammalian gametes through meiosis comprises two subsequent cell divisions. The first division, meiosis I, features highly specific chromosome structures, and behavior, and requires distinct sets of chromosome-associated proteins. Cohesin proteins, of which some are meiosis-specific, are essential for meiosis, but their particular roles in meiosis are incompletely understood. We show here that SMC1β, a meiosis-specific cohesin, serves key functions already in prophase of meiosis I: SMC1β contributes to keeping sister chromatids in cohesion at their centromeres and supports synapsis of the four sister chromatids present in these cells. SMC1β is required for the synapsis of the X and Y sex chromosomes. The failure of autosomes to properly synapse in absence of SMC1β causes extensive alterations in gene expression. This leads to expression of sex chromosome-linked genes, which are lethal at this stage, explaining the death of spermatocytes in mid-prophase I. Together with the analyses of other cohesin proteins and of phosphorylated forms of SMC3 and SMC1α, this paper describes hitherto undescribed properties and functions of meiotic cohesin in sister chromatid cohesion and synapsis.

## Introduction

Meiosis requires unique chromosome structures and dynamics, which are most prominent in the first of the two meiotic divisions (for reviews on aspects of meiosis relevant to this study see [1], [2], [3], [4], [5], [6], [7], [8], [9]. During premeiotic replication, two pairs of sister chromatids are formed from the two homologous chromosomes. Within each pair, the two sister chromatids are linked through sister chromatid cohesion (SCC). In early meiotic prophase I the two pairs of sister chromatids form axial elements (AEs) through association with proteins like SYCP2 and SYCP3. The AEs start to pair and synapse, and full synapsis is reached in pachynema. The synaptonemal complex (SC) is generated, which in addition to AEs includes transverse elements made of SYCP1, SYCE1 and other proteins. Consequently, SYCP1 serves as a marker for synapsis. Other proteins such as HORMAD1 are displaced from chromosomes upon synapsis and thus their association with chromosomes indicates asynapsed chromosomes or chromosomal regions [10], [11]. We use the terms “asynapsed” for never synapsed, and the term “desynapsed” for lost synapsis.

Homologous pairing requires programmed DNA double-strand breaks (DSBs), generated by the type II topoisomerase-like enzyme SPO11. SPO11-deficient mice of both sexes are infertile and *Spo11^−/−^* spermatocytes die in late zygonema/early pachynema. In *Spo11^−/−^* meiocytes, these DSBs are not introduced into DNA, and homologous synapsis of the AEs, which still form, is defective. Non-homologous associations between multiple pairs of sister chromatids are observed [12], [13]. DSBs may give rise to cross-overs and chiasmata. The processing of DSBs requires recombinases RAD51 and the meiosis-specific DMC1. Characteristic RAD51 and DMC1 foci are formed during leptonema and early zygonema at sites of DSBs and disappear progressively during zygonema and early pachynema as repair proceeds.

In spermatocytes, the X and Y sex chromosomes behave uniquely, for they are mostly non-homologous and only synapse at a short, about 700 kbp homologous region called the pseudoautosomal region (PAR) [14], [15]. Within the PAR, meiotic recombination and chiasma formation take place [16], although repair foci appear later on PAR than on autosomes [17]. Compared to synapsed autosomes, a higher density of repair foci and chiasmata is observed at the PAR, probably to ensure chiasma formation in this small region [17]. Asynapsis along most of the sex chromosomes in males has important consequences (reviewed in [9], [18], [19]). The sex chromosomes are packaged into a heterochromatic subnuclear structure called the “sex body” [20], which is enriched for proteins such as HORMAD1 and the ATM/ATR-phosphorylated H2AX, called γH2AX. Gene expression of the asynapsed sex chromosomes is silenced, a phenomenon called meiotic sex chromosome inactivation (MSCI), and H2AX is one of the silencing factors [21].

Gene expression on asynapsed autosomes in leptonema or early zygonema is low [22], and in mutants that fail to completely synapse their autosomes in pachynema, these asynapsed chromosomes or chromosomal regions are silenced as well. This process, named meiotic silencing of asynapsed (or “unsynapsed”) chromosomes (MSUC) [9], [23], [24], [25], depends on proteins like γH2AX. When autosomes are unsynapsed, they accumulate silencing factors at the expense of the sex chromosomes, leading to derepression of X- and Y-linked genes such as *Zfy1/2*, which causes spermatocyte death [26]. In wild-type, upon completion of synapsis in late zygonema/early pachynema, autosomal gene expression is greatly up-regulated [22].

Cohesin is essential for SCC in mitosis and meiosis (for recent reviews see [27], [28], [29], [30], [31], [32]. The cohesin complex consists of a V-shaped heterodimer of two Structural Maintenance of Chromosome proteins, SMC1 and SMC3, whose open ends can be closed by a kleisin protein. A single kleisin, RAD21 (SCC1, MCD1), is expressed in somatic cells, and two meiosis-specific kleisins, REC8 and RAD21L, are additionally expressed in meiocytes, increasing the possible number of cohesin complex variants (reviewed in [33], [34]). Other proteins associate with cohesin, most notably one of three variants of stromal antigen proteins (SA1 to SA3), of which SA3 (STAG3) is meiosis-specific.

Earlier, we reported the identification of a meiosis-specific SMC1 cohesin named SMC1® [35]. SMC1® is detected in spermatocytes starting in leptonema, accumulates along the AEs and SCs in prophase I, and remains at spermatocyte centromeres until the metaphase II-anaphase II transition. SMC1β-deficient meiocytes feature profoundly shortened AEs with extended chromatin loops, telomere aberrations, and partial asynapsis [36]. *Smc1β^−/−^* spermatocytes die in early/mid-pachynema (stage IV of the seminiferous epithelial cycle). In young mice many *Smc1β^−/−^* oocytes survive until metaphase II and show partial loss of SCC or chiasmata in metaphase I. This loss increases with age [37]. At metaphase II no SCC exists anymore in *Smc1β^−/−^* oocytes, which then die. Overall, SMC1β is more abundant in meiocytes than the canonical SMC1α. SMC1α is present on meiotic chromosomes in prophase I, but disappears afterwards. It is unknown whether SMC1α- and SMC1β-type cohesin complexes, or only one of them, provide SCC in prophase I.

Cohesin executes additional functions besides in SCC. In somatic cells cohesin is required for efficient DSB repair through homologous recombination. Cohesin is recruited to sites of DNA damage, and its SMC proteins become phosphorylated in response to DNA damage (reviewed in [38], [39], [40], [41]. Cohesin also regulates gene expression (reviewed in [42], [43], [44], [45] and is thought to promote chromosome looping to support promoter-enhancer interactions. Cohesin may assist the CTCF-mediated insulator function, and often binds to sites that overlap with CTCF binding sites, or to binding sites of other factors.

Together, it remained elusive whether SMC1β contributes to SCC in early meiosis, whether SMC1β plays a particular role in sex body formation and MSCI, whether it acts in induction and processing of DNA DSB repair foci, and whether it affects meiotic gene expression. Our analyses of these features elucidate the relationship between SMC1β and SMC1α, and reveal a consistent concept of a major function of SMC1β in two fundamental processes, SCC and synapsis.

## Materials and Methods

### Mice

*Smc1β^−/−^* and *Spo11^−/−^* mice have been previously described [13], [36], [46] and are in the C57BL/6 background. Ethics statement: Animals were bred and maintained under pathogen-free conditions at the Experimental Center of the Medizinisch-Theoretisches Zentrum of the Medical Faculty at the Dresden University of Technology according to approved animal welfare guidelines, permission number 24-9168.24-1/2010-25 granted by the State of Saxony.

### Antibodies and immunofluorescence

Surface-spread chromosomes were prepared by detergent spreading adapted from [47]. 10 µl of a testicular cell suspension were added to 80 µl 1% Lipsol solution and spread on a glass slide. After swelling of cells for 10 min, 90 µl Peter's fixative (1.25% glutaraldehyde, 1% paraformaldehyde in 0.1 M cacodylate buffer, pH 7.2, containing 0.15% Triton-×100) were added and cell nuclei were allowed to dry for 90 min in a wet chamber. Alternatively, in some experiments, 1.5 µl of single cell suspension were dropped into 7 µl of 0.25% of NP40 on a glass slide. Cells were allowed to lyse for 2 mins and then fixed by adding 24 µl of fixative (1% paraformaldehyde, 10 mM sodium borate buffer pH 9.2). Samples were incubated for 1 h at room temperature in a wet chamber. Object slides were washed twice for 1 min with 0.4% Agepon (AgfaPhoto) and twice for 1 min with water before freezing at −80°C or further usage. For SUMO-1 immunolabeling, spread nuclei of testes were prepared according to Peters et al., 1997 [48]. For immunostaining, cells were permeabilized in 0.5% Triton X-100/PBS for 30 min, quenched in 0.5% glycine/PBS for 5 min, both at room temperature, blocked in PBTG (0.1% BSA, 0.5% fish gelatine, 0.05% Tween-20) for 5 min before primary antibody incubation in PBTG for 90 min at 37°C or overnight at 4°C. Antibodies were used as follows: mouse anti-SYCP3 (hybridoma supernatant), guinea-pig anti-HORMAD1 (1∶100, a kind gift from Dr. A. Tóth, TUD, Dresden, Germany), anti RAD21L (a kind gift from Dr. T. Hirano, Riken, Japan; [49]), γH2AX (1∶100, Upstate, 05-636), SUMO-1 (1∶200 Cell Signaling Technology, #4930), rabbit anti-SMC3 (1∶100), rabbit anti-STAG3(1∶100), rabbit anti SMC1α (1∶20) raised against the C-terminus, rabbit and rabbit or mouse monclonal anti SMC1β as described [36], [50].

### Fluorescence In Situ Hybridization

FISH was performed according to the manufacturer's protocol (Metasystems GmbH). Ten µl of probe mixture was put on slides and covered with a coverslip. Both sample and probe were denatured simultaneously by heating on a hotplate at 75°C for 2 min, followed by incubation of slides in a humidified chamber at 37°C overnight for hybridization. Post hybridization washes were performed with 0.4× SSC at 72°C for 2 min and 2× SSC, 0.05% Tween-20 at room temperature for 30 sec. Slides were incubated with DAPI and signals were analyzed. Cells were identified based on their DAPI staining and staged according to their number of pericentric heterochromatin domains. Wt and *Smc1β^−/−^* pachytene cells were characterized by 5–7 pericentric heterochromatin domains [51], [52].

### Measurement of SPO11–oligonucleotide complexes and western blot analysis

For measurement of SPO11–oligonucleotide complexes, both testes from each mouse were used per experiment, that is, littermate comparisons were made on a per-testis basis. Testis extract preparation, immunoprecipitation and western blot analysis were performed essentially as described [53]. Testes were decapsulated, then lysed in 800 µl lysis buffer (1% Triton X-100, 400 mM NaCl, 25 mM HEPES-NaOH at pH 7.4, 5 mM EDTA). Lysates were centrifuged at 100,000 rpm (355,040× g) for 25 min in a TLA100.2 rotor. Supernatants were incubated with anti-mouse SPO11 antibody 180 (5 µg per pair of testes) at 4°C for 1 h, followed by addition of 40 µl protein-A–agarose beads (Roche) and incubation for another 3 h. Beads were washed three times with IP buffer (1% Triton X-100, 150 mM NaCl, 15 mM Tris-HCl at pH 8.0). Immunoprecipitates were eluted with Laemmli sample buffer and diluted six-fold in IP buffer. Eluates were incubated with an additional 5 µg anti-mouse SPO11 antibody 180 at 4°C for 1 h, followed by addition of 40 µl protein-A–agarose beads and incubation overnight. Beads were washed three times with IP buffer and twice with buffer NEB4 (New England BioLabs). SPO11-oligonucleotide were radio-labelled at 37°C for 1 h using terminal deoxynucleotidyl transferase (Fermentas) and [α-32P] dCTP. Beads were washed three times with IP buffer, boiled in Laemmli sample buffer, and fractionated on 8% SDS–PAGE. Immunoprecipitates were transferred to a PVDF membrane by semi-dry transfer (Bio-Rad). Radiolabelled species were detected and quantified with Fuji phosphor screens and ImageGage software. For western blot analysis, membranes were probed with anti-mouse SPO11 antibody 180 (1∶2,000 in PBS containing 0.1% Tween 20 and 5% non-fat dry milk), then horseradish-peroxidase-conjugated protein A (Abcam; 1∶10,000 in PBS containing 0.1% Tween 20 and 5% nonfat dry milk), and detected using the ECL+ reagent (GE Healthcare).

### Isolation of RNA

Total testis RNA was extracted from four pairs of 12-day-old and 16-day-old *Smc1β^−/−^* and wild-type control littermate mice with TRIzol reagent (Invitrogen Inc.) according to the manufacturer's protocol. Briefly, testes were surgically removed and the Tunica albuginea was detached. Testes were dounce-homogenized in TRIzol reagent prior to phenol-chloroform extraction of RNA. The integrity of the RNA solubilized in water was confirmed by use of the BioAnalyzer (Bio-Rad).

### Microarray procedures

Microarray experiments were performed in quadruplicates with littermate mouse pairs. 800 ng of RNA were applied to microarray analysis on the One-Color Microarray-Based Gene Expression Analysis System (Agilent Technologies Inc.) according to the manufacturer's protocols. Briefly, cDNA was synthesized using M-MLV Reverse Transcriptase followed by labeling with the fluorescent dye Cy3. Amplified cRNA was purified with RNeasy mini spin columns (Qiagen Inc.) followed by hybridization to 4×44K mouse whole genome microarrays (Agilent Technologies). Microarray experiments were performed in quadruplicates with littermate mouse pairs. Scanning of the chips was performed using the Agilent Microarray Scanner. Normalization of the data was performed using the software GeneSpring GX 7.3 (Agilent Technologies). The normalization algorithm was scaling to the median of all samples. No baseline transformation was performed.

### Quantitative reverse-transcription PCR

Total testis RNA of *Smc1β^−/−^* and wt mice was DNase-treated (1.25 U DNase/µg RNA) for 30 min at 37°C and reversely transcribed using Superscript II Reverse Transcriptase (Invitrogen). For PCR amplification, full-length mRNA sequence primer pairs were designed using the web-based tool Primer3 version 3.0 [54] to yield 120 to 180 bp long intron-spanning PCR products. For each reaction, 1 µl of diluted cDNA generated from 40 ng of RNA was amplified in a 10 µl reaction volume using the QuantiTect SYBR Green PCR kit (QIAGEN) in a Rotorgene 3000 thermal cycler (Corbett Research Inc.). Reactions were performed in duplicates and two or three mouse pairs were analyzed for each gene. Average mRNA levels of beta-actin (*Actb*) and glyceraldehyde-3-phosphate dehydrogenase (*Gapdh*) were used for normalization. PCR primer sequences are shown in Supplementary Table S1.

### Spatial clustering of differentially regulated genes

#### 1. Using the Wilcoxon test

Chromosomal regions enriched for up- or down-regulated genes were identified using the two-sided Wilcoxon rank sum test [55], as implemented in R (function wilcox.test.R). A 10-gene sliding window approach was used, testing if genes inside the given window are more strongly up- (or down-) regulated than expected by chance. Unlike the t-test, the non-parametric Wilcoxon test makes no assumptions about the distribution of the data. In order to quantify the global significance (accounting for multiple testing) fold-changes were permuted 10 times across the whole genome and the same approach was applied again. Observed Wilcoxon scores were compared to the permutation-based scores in order to determine False Discovery Rates (FDR). Reported clusters have a FDR less than 0.2.

#### 2. Using Hidden Markov Models

Average log-ratio profiles of gene expression levels for *Smc1β^−/−^* compared to wt testes have also been analyzed using a three-state Hidden Markov Model (HMM) with Gaussian emission distributions as described [56]. Hidden states were determined using a Bayesian Baum-Welch algorithm: decoding of the most likely expression state of each gene (under-expressed, unchanged, or over-expressed in *Smc1β^−/−^*) has been done using state-posterior decoding assigning each gene to its most probable underlying expression state. Groups of contiguous genes identified as underexpressed or groups of contiguous genes identified as being overexpressed where considered as domains. Applications of the HMM to permuted log-ratio profiles (100 permutations per chromosome) identified that domains of length greater or equal than two have been observed significantly more frequently in the original data than in the permuted profiles.

### MiRNA expression profiling

MiRNA expression analysis was performed using the miRXplore microarrays (Miltenyi Biotech GmbH) according to the manufacturer's protocol. Microarrays carried DNA oligonucleotides with a reverse-complementary sequence of mature mouse miRNAs. The 50th percentile of background intensity values was applied for data normalization and dye bias was corrected by Lowess normalization. The normalized mean ratios of *Smc1β^−/−^* versus wt were calculated.

The microarray mutant versus wild-type log fold changes were normalized according to the more stable RT-qPCR measurements of 20 selected genes. The respective regression model, which was applied to all the genes, is the following. Y corresponds to the RT-qPCR measurement and x to the microarray fold change. The normalized data have been subjected to statistical clustering testing by applying a two-sided Wilcoxon rank sum test [55] as implemented in R (function wilcox.test.R). A 10 gene sliding window approach was used, testing the null hypothesis that the fold changes of the genes in each window cluster better than in the rest of the genome. Application of the same approach to permuted log-ratio profiles (10 whole genome permutations) identified that the detected clusters have been observed significantly more frequently in the orignal data than in the permuted ones. The respective false discovery rates (FDR) are plotted in dots across chromosomes 1-X.

### Fluorescence-activated cell sorting (FACS)

Single-cell suspensions of testes were resuspended in FACS buffer (HBSS supplemented with 20 mM HEPES (pH 7.2), 1.2 mM MgSO_4_, 1.3 mM CaCl_2_, 6.6 mM sodium pyruvate, 0.05% lactate, glutamine, and 1% fetal calf serum) at a density of 2 million cells/ml. Bis-benzamide Hoechst33342 (5 µg/ml, Hoechst) was added before incubation of the cells for 1 h at 32°C. For exclusion of dead cells, propidium iodide (PI, 2 µg/ml) was added before FACS analysis. Cells were analyzed using a LSRII flow cytometer (BD Biosciences) using the FACSDiva software version (BD Biosciences, version 6.1.3). Hoechst was excited with the 355 nm UV laser and emission filters Emerald (585/42 nm, LP) and Alexa 350 (505 nm) were applied. PI was excited with the 488 nm blue laser and emission was filtered using PE (685 nm).

## Results

### Autosomal asynapsis and defective sex body formation in *Smc1β^−/−^* spermatocytes

The current study aimed at elucidating in detail the role of SMC1β in SCC and synapsis, and therefore a number of meiotic processes and features were analyzed, all of which relate to these two processes.

Previously, we showed that loss of SMC1β causes partial asynapsis of many autosomes and total asynapsis of a few [36], [57]. In wild-type (wt) pachytene cells only the sex body with its large unpaired regions stains intensely for γH2AX. In *Smc1β^−/−^* spermatocytes that we classified as early/mid pachytene small clouds of γH2AX staining were observed on many chromosomes [36]. The most advanced *Smc1β^−/−^* spermatocytes show widespread SYCP1 staining, little HORMAD1 staining, and a very low percentage of cells express H1t, a mid-pachytene marker. We classified the early/mid pachytene *Smc1β^−/−^* spermatocytes based on the highest degree of axes compaction that can be observed. The sex body was mostly absent *Smc1β^−/−^* spermatocytes, and if a prominent γH2AX cloud was seen, it was one among several γH2AX clouds, and thus the occurrence of a sex body remained uncertain.

**Figure 1:**
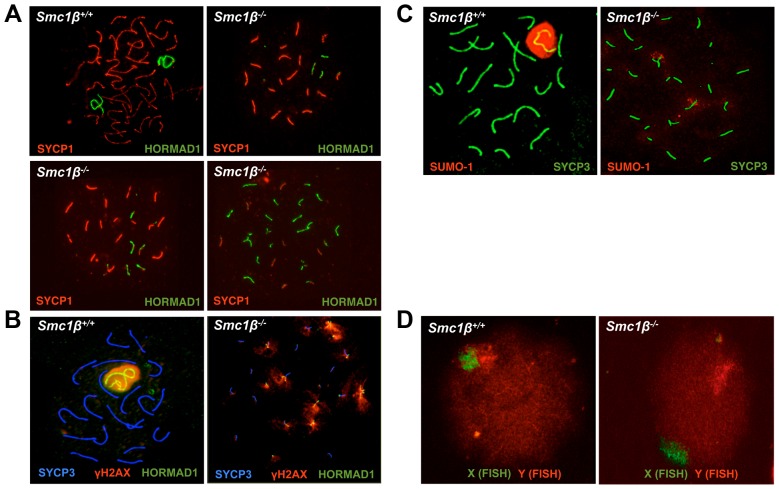
Synapsis defects of autosomes and sex chromosomes of *Smc1β^−/−^* spermatocytes. **A**. Wt and *Smc1β^−/−^* spermatocyte chromosome spreads stained with anti SYCP1 for synapased chromosomes and anti HORMAD1 for unsynapsed chromosomes or chromosomal regions. Three examples of *Smc1β^−/−^* cells illustrate different degrees of asynapsis (n = 60). **B**. Staining of the sex body in wt and *Smc1β^−/−^* spermatocyte chromosome spreads using anti SYCP3 for AEs, anti γH2AX for the sex body or unsynapsed chromatin domains, and anti HORMAD1 for the unsynapsed sex chromosome axes or unsynapsed autosomes. **C**. Staining of the sex body in wt and *Smc1β^−/−^* spermatocyte chromosome spreads using anti SYCP3 for AEs, and anti SUMO-1 for the sex body or unsynapsed chromatin domains. **D**. FISH of wt (n = 40) and *Smc1β^−/−^* (n = 60) spermatocytes using a green (X) and red (Y) sex chromosome-specific probe.

To initiate the present study, a thorough analysis of spermatocyte asynapsis was performed using various combinations of staining for SYCP1, SYCP3, γH2AX, HORMAD1, and SUMO-1 (Fig. 1, Suppl. Fig. S1). SYCP1 localizes to synapsed regions of prophase I chromosomes, SYCP3 stains asynapsed and synapsed axial elements (AEs), and γH2AX localizes to asynapsed regions, which are also transcriptionally silenced regions, and to DNA DSBs. HORMAD1 specifically stains asynapsed chromosomes or asynapsed regions of chromosomes and disappears from chromosomes upon formation of the SC [10], [11], and SUMO-1 localizes preferentially to asynapsed regions including the sex body [58], [59], [60]. The level of asynapsis varies considerably between individual *Smc1β^−/−^* cells as illustrated in Fig. 1A, but there were virtually no early to mid pachytene *Smc1β^−/−^* spermatocytes without at least partial asynapsis. The average number of partially asynapsed chromosomes was 1.8+/−0.9 per cell, the average number of entirely asynapsed chromosomes including the sex chromosomes was 6.8+/−1.7 per cell. Except the unsynapsed regions of the X and Y chromosomes, there are no asynapsed chromosomes in wt pachytene cells.

Synapsis of X and Y chromosomes is restricted to the short PAR. In the vast majority of *Smc1β^−/−^* spermatocytes neither the PAR-synapsed X and Y chromosomes nor the typical sex body was detected by either HORMAD1, γH2AX, or SUMO-1 staining combined with labeling the SC (SYCP1) or the AEs (SYCP3) (Fig. 1B, 1C). HORMAD1, γH2AX or SUMO-1 localized to several asynapsed chromosomes. Quantification of γH2AX staining revealed that a typical sex body could not be observed. Rather, two (app. 25% of the cells) or more (app. 72% of the cells) γH2AX clouds or multiple patches, of which occasionally (app. 3% of cells) one was more prominent, were found. In addition, we also evaluated X-Y pairing using fluorescence in situ hybridization (FISH) (Fig. 1D). Pachytene cells were identified by light microscopy based on their characteristic DAPI-stained pattern of heterochromatin [51], [52] (Suppl. Fig. S1D). In 98% of the *Smc1β^−/−^* cells (59 of 60), the two FISH signals were clearly apart, indicating that the X and Y were not synapsed within the PAR, consistent with the absence of a sex body in which both chromosomes ought to be present. In one of the cells the signals were very near each other, but FISH did not allow us to determine if PAR synapsis had occurred. In wt, 90% of the cells (36 of 40) showed partially overlapping signals, and in the remaining four cells, the signals did not visibly overlap but were very close to each other.

This indicates that X/Y synapsis and sex body formation fails in the absence of SMC1β, and that the remaining SMC1α-type cohesin complexes alone are not sufficient for either process. This also implies that *Smc1β^−/−^* spermatocytes contain at least 21 microscopically visible chromosomes, i.e. at least 19 fully or partially formed SCs and the two separate sex chromosome AEs. Thus every cell has at least one pair of fully asynapsed chromosomes – the X and Y chromosomes –, and often many more, in addition to some partial asynapsis.

### Disturbed patterns of gene expression in *Smc1β^−/−^* spermatocytes

The previous section showed significant levels of autosomal asynapsis and a failure to synapse the X and Y chromosomes in absence of SMC1β. High levels of autosomal asynapsis correlate with decreased expression of autosomal genes, i.e. MSUC, and with decreased MSCI. In male mice with mutations in genes essential for establishment of synapsis (*Spo11, Dnmt3l, Msh5, Dmc1*), regions of asynapsis are transcriptionally silenced through sequestration of silencing factors such as γH2AX that are no longer available to establish MSCI [24]. The deficiency to form sex bodies in *Smc1β^−/−^* spermatocytes, and thus the failure to see γH2AX accumulation on the sex chromosomes, suggests failing MSCI. In addition, the presence of autosomal asynapsis in *Smc1β^−/−^* spermatocytes renders an MSUC response likely. However, a direct contribution of cohesin to regulation of gene expression in meiotic cells appears also possible considering this role of cohesin in somatic cells. To test this, we performed microarray analysis of gene expression of wt and *Smc1β^−/−^* spermatocytes. We chose to compare mRNA expression in testes from 16 day-old mice when the first, synchronized wave of meiosis proceeds. At this stage the cells in our wt and *Smc1β^−/−^* littermate mice have just reached pachynema. There is no apoptosis yet in *Smc1β^−/−^* mice, where apoptosis commences at day 17 at the earliest [36]. Minor differences were observed in the cellularity of wt and *Smc1β^−/−^* testes at that age (Suppl. Fig. S2).

**Figure 2:**
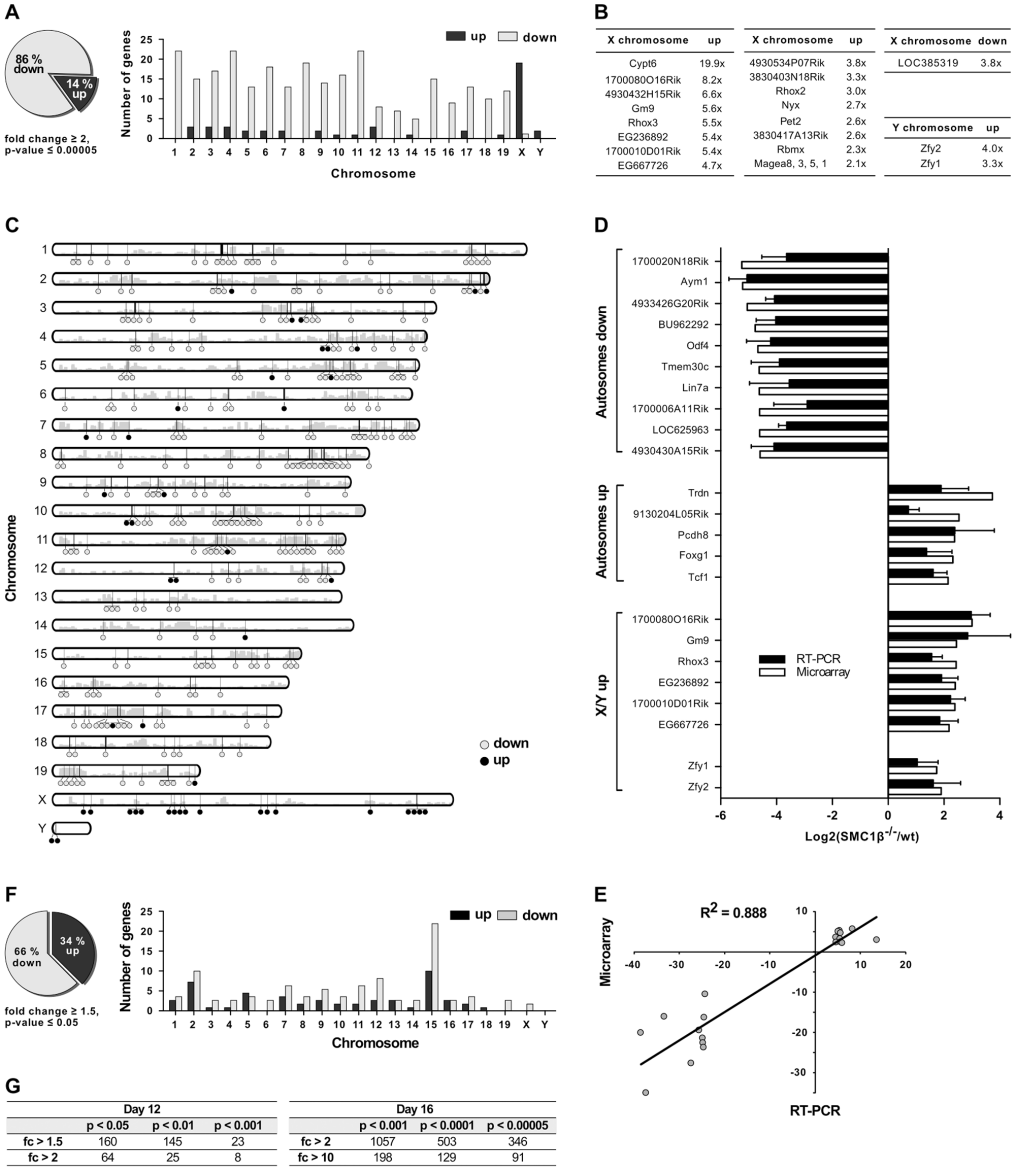
Gene expression analysis of testes of juvenile *Smc1β^−/−^* mice. **A–E.** Analysis of total testis gene expression patterns of *Smc1β^−/−^* mice aged 16 dpp compared to wt littermates. **A**. In testes of *Smc1β^−/−^* mice, 86% of genes changed least two-fold in expression are down-regulated (grey) and 14% are up-regulated (black, left panel). Mis-expressed genes are predominantly down-regulated on the autosomes and mostly up-regulated on the X and Y chromosomes (right panel). **B**. Genes on X and Y chromosomes with highest fold changes in microarray-based profiles. **C**. Idiogram of chromosomal location of mis-expressed genes. Down-regulated (grey) and up-regulated (black) genes are represented by circles and gene density is depicted by grey bars. **D**. Microarray-based expression of 21 selected genes was validated by quantitative reverse transcriptase PCR (RT-PCR). Genes were grouped according to chromosomal location and the mode of regulation. **E**. Microarray-based fold changes of the 21 selected genes correlate with RT-PCR data. The coefficient of determination R^2^ = 0.888 confirms high correlation. **F**. In testes of *Smc1β^−/−^* mice aged 12 dpp, 66% of genes changed in expression by at least 1.5-fold are down-regulated (grey) and 34% are up-regulated (black, left panel). Mis-expressed genes are broadly distributed amongst chromosomes and moderately enriched on chromosome 15 (right panel). **G**. Numbers of mis-expressed genes in testes of *Smc1β^−/−^* mice aged 12 dpp (left) and 16 dpp (right) with different statistical significance and fold regulation thresholds.

Gene expression patterns of testes of four pairs of wt and *Smc1β^−/−^* littermates were analyzed. We performed eight independent gene expression measurements using the One-Color 4×44K Whole Mouse Genome Array system (Agilent). The data yielded a highly significant pattern of changes in gene expression (Fig. 2A). Of all 347 genes whose expression was altered in *Smc1β^−/−^* spermatocytes by at least two-fold with a stringent p-value threshold of 5×10^−5^, 85% were down-regulated and 15% up-regulated. Of the aberrantly regulated autosomal genes, 91.2% were down-regulated, between 5 and 25 genes per chromosome (Fig. 2). In striking contrast, a large number of up-regulated genes are found on the X chromosome. Nineteen X chromosomal genes were up-regulated, while only 2 X-linked genes were down-regulated (Fig. 2B). On the Y chromosome, 2 genes were up-regulated, none were down-regulated. Thus, early pachytene spermatocyte gene expression on autosomes and sex chromosomes reacts inversely to loss of SMC1β.

For the validation of microarray data, reverse transcription quantitative PCR (RT-qPCR) was performed for top hits of several categories of mis-regulated genes. The genes were grouped into autosomal down-regulated genes, autosomal up-regulated genes and mis-regulated genes on the X- and Y-chromosome. For 21 of the genes of these groups, expression ratios from microarrays and RT-qPCRs from two or three mouse pairs are depicted in Figure 2D. Respective ratios of data from the microarray and RT-qPCR analyses correlated with a high correlation coefficient (R^2^ = 0.88) (Fig. 2E).

Interestingly, the Y-linked *Zfy1/2* genes, which were found up-regulated in *Smc1β^−/−^* spermatocytes, are known to strongly contribute to the pachynema, stage IV, quality surveillance mechanisms [26]. Expression of the transcription factor ZFY1/2 at that stage causes cell death. Up-regulation of *Zfy1/2* gene expression was also confirmed by RT-qPCR (Fig. 2D) and suggests that this contributes to apoptosis of stage IV *Smc1β^−/−^* spermatocytes.

To test if certain genomic regions in *Smc1β^−/−^* pachytene cells show specific clustering of up- or down-regulated genes we performed two types of analysis, using (1) a two-sided Wilcoxon rank sum test using a sliding window of 10 genes, and (2) a three-state Hidden Markov model (Fig. 2C; Suppl. Fig. S3, S4). Both methods identified focal clusters of predominantly down-regulated genes on the autosomes, whereas clusters consisting of primarily up-regulated genes were identified on the X-chromosome (the Y-chromosome was excluded from this analysis). However, after correcting for multiple hypothesis testing, the remaining number of significant clusters on the autosomes was relatively small because of the global down-regulation on the autosomes. Thus, due to the large number of down-regulated genes on the autosomes such clusters could theoretically emerge by chance.

The patterns of changes in gene expression between leptonema and mid-pachynema are complex and not yet understood at high resolution for individual, defined stages or cell populations. Generally, it is thought that with completion of synapsis in pachynema, autosomal gene expression increases as the silencer proteins such as γH2AX retract from synapsed autosomes and assembles on the sex chromosomes. In early/mid pachytene *Smc1β^−/−^* spermatocytes a significant number of autosomal genes fails to be expressed. To determine whether the down-regulation of autosomal gene expression seen in *Smc1β^−/−^* spermatocytes correlates with synapsis, i.e. likely originates from an MSUC response rather than a direct effect of SMC1β on transcription, we prepared mRNA from testes of 12 day-old mice (Fig. 2F). At that stage, spermatocytes of our mouse strains are in early zygonema and thus there is still little synapsis. If SMC1β has a direct, synapsis-independent effect on transcription it should be measurable at this stage, similar to the pachytene effect, albeit limited to those relatively few genes that are normally expressed in early zygonema [22]. Assaying the samples by the same microarray technology revealed, however, not a single down- or up-regulated gene at a p-value below 0.0001 and a minimum 2-fold change (Fig. 2F, 2G). At a less stringent p-value of 0.001 eight genes were identified of which half were down-regulated, half up-regulated. In contrast, at this p-value 1080 genes, almost all down-regulated, were identified for the pachytene samples. This strongly suggests a direct correlation of autosomal down-regulation with the failure to completely synapse. Chromosomal distribution analysis of 160 mis-regulated genes (day 12) identified at low stringency (p<0.05; fc>1.5) shows them on all chromosomes (Fig. 2F). Many of the mis-regulated genes localize to chromosome 15 with an enrichment in the proximity to the *Smc1β* locus, indicative of an artifact generated through alterations of the locus environment by gene targeting. We therefore disregarded these genes. No significant changes were detected in miRNA microarrays (Suppl. Fig. S5).

Thus the vast majority of down-regulation in *Smc1β^−/−^* pachytene cells is very likely not caused by a direct effect of SMC1β on transcription, but rather by an MSUC response.

### Phosphorylated SMC1 and SMC3 distinctly localize to the sex body

Recently it was shown that synapsis affects localization of phosphorylated SMC3 [61]. In mitotic cells, SMC1 and SMC3 become phosphorylated in an ATM- or ATR-dependent manner within the DNA damage response [62], . SMC1 and SMC3 localize to DSBs in somatic cells (reviewed in [40], [64]), and in this respect behave similar to γH2AX. ATR localizes to asynapsed regions in meiocytes [9], and recently it was reported that pSer1083-SMC3 also localizes preferentially to asynapsed chromosomes, including the sex chromosomes in pachynema and diplonema [61]. We extended this observation, and asked whether pSer966-SMC1 also localizes to asynapsed regions including the sex body, and how phosphorylated SMC1α and SMC3 behave in mutant backgrounds.

**Figure 3:**
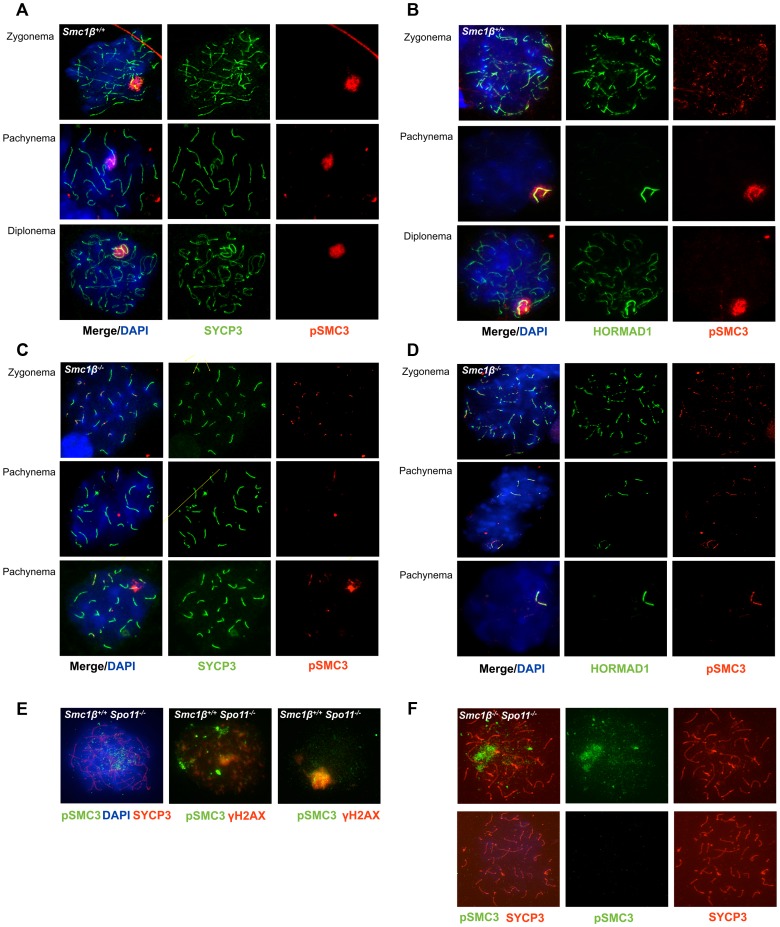
Phosphorylated SMC3 distinctly localizes to asynaptic regions in wt, *Smc1β^−/−^*, and *Smc1β^−/−^Spo11^−/−^* spermatocytes. **A**. Wt spermatocyte chromosome spreads stained with anti SYCP3 for AEs and anti phosphorylated SMC3(S1083). **B**. Staining of unsynapsed chromosome axes and of sex chromosomes axes in wt spermatocyte spreads with anti HORMAD1, combined with staining by anti phosphorylated SMC3(S1083). **C**. Staining of *Smc1β^−/−^* spermatocyte chromosome spreads using anti SYCP3 for AEs and anti phosphorylated SMC3(S1083). **D**. Staining of unsynapsed sex chromosome axes or unsynapsed autosomes in *Smc1β^−/−^* spermatocyte chromosome spreads with anti HORMAD1, combined with staining by anti phosphorylated SMC3. **E**. Staining of *Spo11^−/−^* spermatocyte chromosome spreads using anti phosphorylated SMC3(S1083) combined with either anti SYCP3 and DAPI (left, top), or with anti γH2AX (n = 40). **F**. Staining of *Smc1β^−/−^Spo11^−/−^* spermatocyte chromosome spreads with anti SYCP3 and anti phosphorylated SMC3(S1083) (n = 190).

In leptonema and early zygonema, pSer1083-SMC3 localizes along chromosomes in a punctuated pattern, in pachynema and diplonema pSer1083-SMC3 marks the sex body (Fig. 3A). Unlike HORMAD1, which localizes along the sex chromosome axes (Fig. 3B), pSer1083-SMC3 appears as a cloud similar to, but not as expanded as the γH2AX cloud (Fig. 3A, B and Fig. 1B). In wt pSer1083-SMC3 is found exclusively at the sex body from late zygonema to diplonema (Fig. 3A). Controls showed that the phospho-SMC1 peptide did not block anti pSer1083-SMC3 reactions, but the phosphorylated SMC3 epitope peptide eliminated the signal (Suppl. Fig. S6A). No X/Y axes staining appeared even at low concentrations of the pSMC3 peptide, i.e. the pSer1083-SMC3 always appeared as a cloud. In metaphase I, pSMC3 was observed at centromeres, a prominent structure stained by SYCP3 at this stage (Suppl. Fig. S6B).

In *Smc1β^−/−^* spermatocytes (Fig. 3C, D) pSer1083-SMC3 localizes to unsynapsed chromosomes in late zygotene. In early/mid pachytene the pSer1083-SMC3 is not detected at sex bodies, which as described above do not form. Occasionally, deposits of pSer1083-SMC3 are seen that associate with part of a chromosome. On *Smc1β^−/−^* spermatocyte spreads the pSer1083-SMC3 is observed at the HORMAD1-stained asynapsed chromosomes, regardless of their number (Fig. 3D). Since SMC1α is present [36] and SMC1β-deficient cells were analyzed, this indicates that an SMC1α-pSer1083SMC3 complex marks asynapsed regions, at least in the absence of SMC1β.

**Figure 4:**
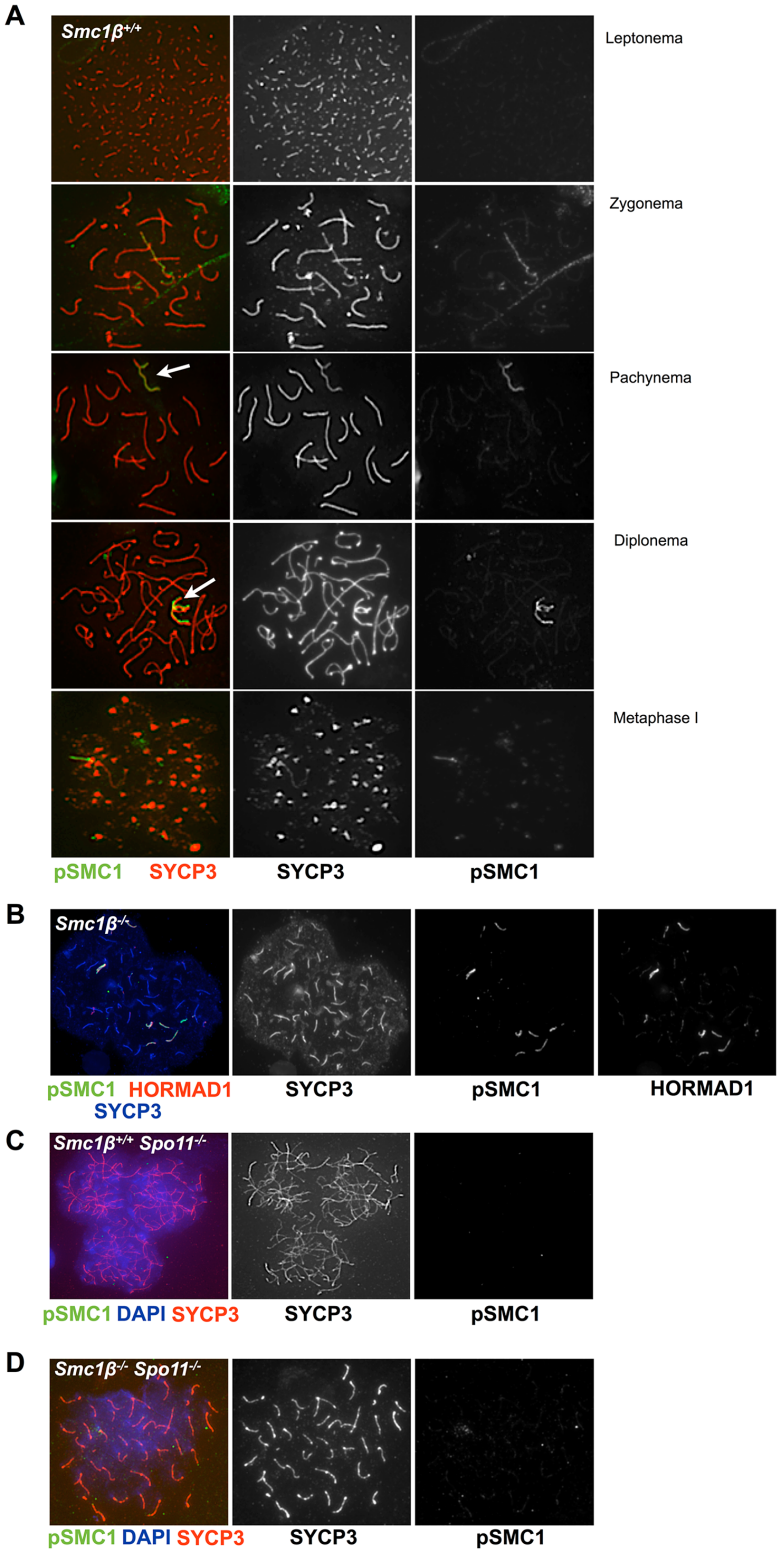
Phosphorylated SMC1α distinctly localizes to asynaptic regions in wt, *Smc1β^−/−^*, and *Smc1β^−/−^Spo11^−/−^* spermatocytes. **A**. Staining of wt spermatocyte chromosome spreads with anti SYCP3 and anti phosphorylated SMC1α(S966) (n = 150). **B**. Staining of *Smc1β^−/−^* (n = 30) spermatocyte chromosome spreads with anti phosphorylated SMC1α(S966), anti HORMAD1 (H1) and anti SYCP3. **C**. Staining of *Spo11^−/−^* (n = 90) and **D**. *Smc1β^−/−^Spo11^−/−^* (n = 120) spermatocyte chromosome spreads with anti SYCP3 and anti phosphorylated SMC1α(S966), and DAPI.

The anti pSer966-SMC1 antibody recognizes only phosphorylated SMC1α since the Ser966 is not conserved between SMC1α and SMC1β. In wt leptonema spermatocytes, no pSer966-SMC1 was detected. In late zygonema, pSer966-SMC1 is preferentially seen at the asynapsed axes. In pachynema and early diplonema pSer966-SMC1 localizes only to the sex body, and in late diplonema pSer966-SMC1 disappears (Fig. 4A). pSer966-SMC1 localizes almost exclusively to the chromosome axes and does not form clouds like pSer1083-SMC3. This suggests that non-phosphorylated SMC1α/pSMC3 complexes localize to the sex chromosome loops and show as clouds, and that pSMC1α/SMC3 complexes associate with the X/Y axes. It is rather unlikely that an alternative SMC1β/pSMC3 complex localizes to the sex body cloud, since SMC1β was only seen at the sex chromosome axis [35]. In *Smc1β^−/−^* spermatocytes, the pSer966-SMC1 localizes along the asynapsed chromosomes (Fig. 4B).

### SPO11-generated DSBs are not required for cohesin loading but for cohesin phosphorylation

Cohesin can be loaded onto mitotic cell chromosomes upon introduction of DNA DSBs [65]. The use of *Spo11^−/−^* mice lacking programmed DSBs [12], [13] allowed us to analyze the recruitment of cohesins, including phosphorylated SMC1 and SMC3, to meiotic chromosomes. Earlier, SMC1α and SMC3 were found to localize to chromosomes of *Spo11^−/−^* spermatocytes [66]. We observed all of the cohesin proteins tested here to localize to chromosomes of *Spo11^−/−^* cells, including SMC1α, SMC1β, SMC3, STAG3, RAD21 and RAD21L (Suppl. Fig. S7A–G). The patterns of association differed between individual cohesins. Costaining of SMC1α with HORMAD1 revealed that in *Spo11^−/−^* spermatocytes SMC1α localizes along AEs preferentially where no HORMAD1 associates, i.e. more SMC1α localizes to regions of non-homologous associations. There is little SMC1α on non-associated chromosomes (Suppl. Fig. S7A; quantification in Suppl. Fig. S8). As published before [50], in wt cells SMC1α localizes along every chromosome, including asynapsed chromosomes in zygonema, and synapsed chromosomes and the unpaired region of the X/Y in pachynema, and stains in comparable intensities whether synapsed or not. In *Spo11^−/−^* spermatocytes SMC3, the only cohesin subunit present in all cohesin complexes, localizes to asynapsed regions decorated with HORMAD1 and to non-homologously synapsed regions (Suppl. Fig. S7B and quantification in Suppl. Fig. S8). In wt, SMC3 also localizes to asynapsed and synapsed regions with equal intensity [50]. SMC1β localizes to the axes of all chromosomes in wt and *Spo11^−/−^* spermatocytes. Together, this suggests that SMC1β and not SMC1α complexes localize preferentially to non-associated regions (Suppl. Fig. S7G). The data also suggest that localization of SMC1α to asynapsed regions depends on SPO11. STAG3 decorates all axes in a punctuate pattern in wt and *Spo11^−/−^* cells (Suppl. Fig. S7C). RAD21L localizes to the asynapsed axes in *Smc1β^−/−^* spermatocytes (Suppl. Fig. S7D). RAD21L also localizes in a somewhat punctated fashion all along the *Spo11^−/−^* axes, and in wt pachytene cells preferentially accumulates at asynapsed regions, particularly the sex chromosomes (Suppl. Fig. S7E). In wt, RAD21 co-localizes in many dots with SYCP3 along autosomes, but there is little overlap with SYCP3 on the sex chromosomes (Suppl. Fig. S7F), where the RAD21 signals accumulate next to the axes. In *Spo11^−/−^* chromosome spreads, very little of RAD21, which appears as many dots throughout the chromatin, co-localizes with SYCP3 on the chromosome axes.

In spermatocytes lacking SPO11 we detected no pSer1083-SMC3 on chromosomes axes, whether unsynapsed or non-homologously associated, consistent with the report by Fukuda et al. (2012) (Fig. 3E). However, we found pSer1083-SMC3 to form some clusters within the nuclei reminiscent of pseudo sex bodies, and to localize in dispersed spots throughout the nuclei in *Spo11^−/−^* spermatocytes (Fig. 3E). In many cells there was overlap of pSer1083-SMC3 with staining for γH2AX at the pseudo sex bodies. In *Smc1β^−/−^Spo11^−/−^* spermatocytes, the localization of pSer1083-SMC3 in many cells is similar to that seen in *Spo11^−/−^* spermatocytes, but in about 40% of the cells, no staining for pSer1083-SMC3 was observed (Fig. 3F). Together this indicates that SMC1α forms complexes with pSer1083-SMC3 in absence of SPO11 and SMC1β. We also noted moderately longer chromosome axes in *Smc1β^−/−^Spo11^−/−^* spermatocyte spreads as compared to *Smc1β^−/−^*spreads (Fig. 4B, 4D; quantification in Suppl. Fig. S9), although this varied and in some cells this difference was small. In *Smc1β^−/−^* spermatocytes the pSer966-SMC1 signal is seen on asynapsed autosomes (Fig. 4B). This supports the above hypothesis of pSMC1α/SMC3 complexes to localize to asynaptic regions. No pSer966-SMC1 was detected in *Spo11^−/−^* or *Smc1β^−/−^Spo11^−/−^* spermatocytes (Fig. 4C, D). These data indicate that SPO11, and presumably SPO11-generated DSBs, are required for SMC1α Ser966 phosphorylation, consistent with its kinetics of appearance in early zygonema.

### Delayed disappearance of repair foci in absence of SMC1β

Asynapsis may have consequences for the processing of meiotic double-strand breaks (DSBs) as suggested by the correlation of synapsis and disappearance of repair foci, which is delayed on the largely unpaired X/Y chromosomes. To address formation and processing of DSBs in *Smc1β^−/−^* spermatocytes, we first determined whether programmed DSBs are properly generated in absence of SMC1β. Thus, SPO11-dependent DSBs were measured by visualizing the SPO11-oligonucleotide intermediate product of the reaction [67].

**Figure 5:**
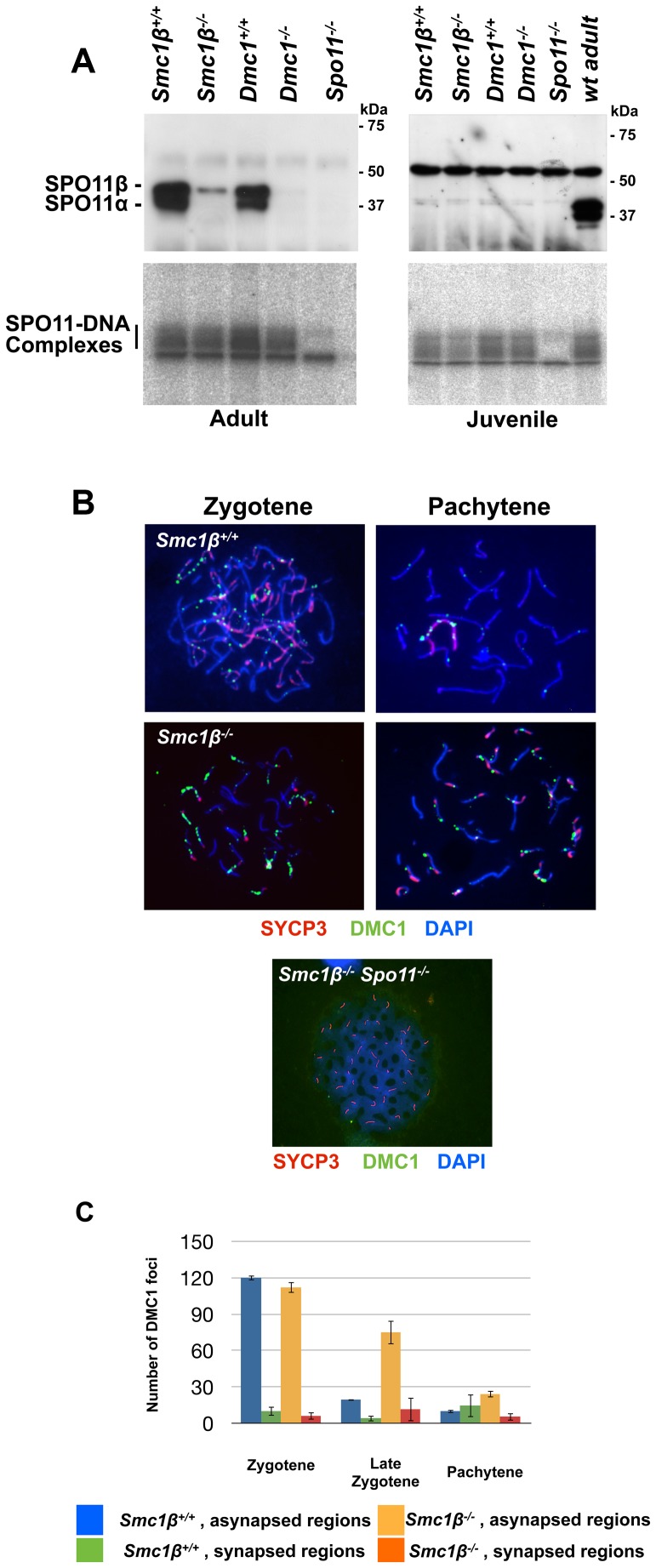
Normal formation but delayed processing of DNA DSBs in *Smc1β^−/−^*spermatocytes. **A**. Top: Immuno blot of nuclear protein extracts from testis of the indicated mouse strains, probed with anti SPO11 antibody. The two SPO11 variants are indicated. Bottom: SPO11-DNA complexes assayed as described in Materials and Methods, analyzed in testis from adult or juvenile (day 14.5 pp) mice of the indicated mouse strains. **B**. Staining of wt (n = 120), *Smc1β^−/−^* (n = 160) or*Smc1β^−/−^Spo11^−/−^* (n = 80) spermatocyte chromosome spreads with anti HORMAD1, anti DMC1 and anti SYCP3, and DAPI. **C** Graphical representation of DSB processing in wt and *Smc1β^−/−^* spermatocytes at the indicated stages of meiosis. Numbers of DMC1 foci in synapsed and asynapsed regions are provided.

Fig. 5A demonstrates that SPO11 protein is produced in adult wt and *Smc1β^−/−^* cells, although in the latter the SPO11α splice variant is absent as it is expressed later and may not contribute much to leptotene spermatocyte DSB formation [68]. The alternative splice isoform SPO11β is present although in much smaller quantities as in wt, probably because late pachytene cells, which contribute significantly to total SPO11 in spermatocytes, are absent. The early SPO11 enzyme is sufficient to produce substantial levels of SPO11-oligonucleotide complexes, which is also seen in the control *Dmc1^−/−^* samples that contain even less SPO11 enzyme, and whose spermatocytes arrest in late zygonema. *Smc1β^−/−^* spermatocytes develop further into pachynema and probably therefore synthesize more SPO11.

We assessed steady-state levels of SPO11-oligonucleotide complexes in whole-testis extracts from wt and mutant mice. We found a very mild reduction in adult *Smc1β^−/−^* testes (0.84±0.20-fold relative to wt, mean and s.d., n = 2 littermate pairs). In contrast, adult *Dmc1^−/−^* testes, which are similarly reduced in size as *Smc1β^−/−^* testes, displayed a more substantial reduction in SPO11-oligonucleotide complex levels (0.40±0.07-fold, n = 2), as previously reported [53]. To account for different sizes and cellularities in wt and mutant testes, we also examined SPO11-oligonucleotide levels in juvenile mice at day 14.5 post partum, when the majority of spermatocytes have not yet progressed far enough into meiotic prophase to be affected by the mid-pachytene checkpoint. At this age, SPO11β is only weakly expressed, but SPO11-oligonucleotide complexes are readily detectable. Complex levels were moderately reduced in *Smc1β^−/−^* testes (0.68±0.14-fold, n = 3) and in *Dmc1^−/−^* testes (0.77±0.03-fold, n = 2). This does not argue for an impairment in SPO11 DSB formation, since minor variations such as slightly delayed initial spermatogenesis in the *Smc1β^−/−^* mutant may cause this phenotype.

Efficient production of DSBs in the adult was confirmed by quantifying the number of initial repair foci. Repair proteins assemble at DSBs and form foci whose appearance is indicative of DSBs, and whose processing, i.e. disappearance, is indicative of proper repair. IF analysis of RAD51 and DMC1 foci revealed that the same number of foci are generated in wt and *Smc1β^−/−^* spermatocytes at the early zygotene stage (Fig. 5B, Suppl. Fig. S10). In late zygotene and early pachytene, these foci are processed and disappear in wt cells, but fail to do so efficiently in *Smc1β^−/−^* spermatocytes. Detailed analysis of the persistent foci by co-staining with HORMAD1 revealed that they localize predominantly to asynapsed regions and to sex chromosomes (Fig. 5B, C). As expected, no foci were detected in *Spo11^−/−^* (not shown) and in *Smc1β^−/−^Spo11^−/−^* spermatocytes (Fig. 5B).

### Non-homologous synapsis requires SMC1β

**Figure 6:**
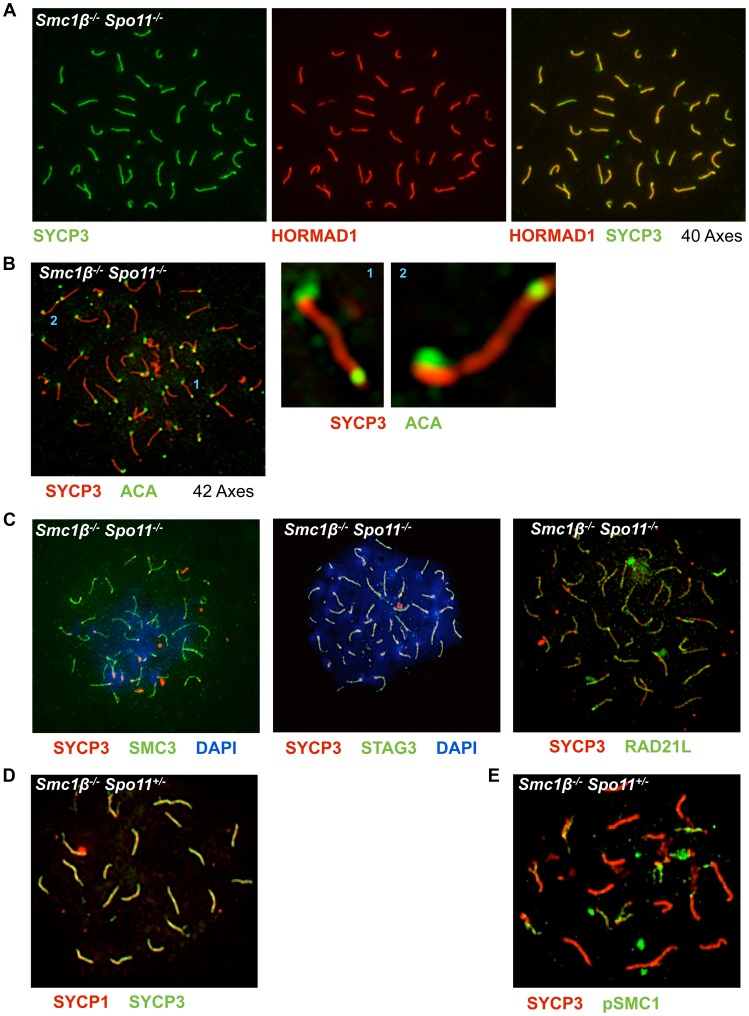
Non-homologous association of axial elements depends on SMC1β. **A** *Smc1β^−/−^Spo11^−/−^* spermatocyte chromosome spreads were stained with anti SYCP3 for AEs and anti HORMAD1 for unsynapsed chromosomes or chromosomal regions (n = 70). **B** *Smc1β^−/−^Spo11^−/−^* spermatocyte chromosome spreads stained with anti SYCP3 for AEs and anti ACA for centromere staining. Two examples where chromosomes apparently show fusion at the centromere-distal ends are shown. **C**. Localization of different cohesin proteins in *Smc1β^−/−^Spo11^−/−^* spermatocyte chromosome spreads, which were stained with anti SYCP3 for AEs, SMC3 (n = 40), STAG3 (n = 30), and RAD21L (n = 30) for different cohesin proteins. **D**. Staining of *Smc1β^−/−^Spo11^+/−^* spermatocytes for SYCP1 and SYCP3. **E**. Staining of *Smc1β^−/−^Spo11^+/−^* spermatocytes for pSer966-SMC1α and SYCP3.

Staining of *Smc1β^−/−^Spo11^−/−^* spermatocyte spreads (Fig. 4D) unexpectedly showed 40 clearly separate, SYCP3-stained chromosome cores. Therefore, we further analyzed whether non-homologous associations in *Spo11^−/−^* spermatocytes depend on SMC1β. Staining of *Smc1β^−/−^Spo11^−/−^* spermatocyte spreads for HORMAD1 and SYCP3 showed 40 separate, short chromosome cores, i.e. univalents without any non-homologous association (Fig. 6A). Axes shortening is a known, prominent phenotype of *Smc1β^−/−^* spermatocytes [36]. Since *Smc1β^−/−^Spo11^−/−^* spermatocytes die slightly earlier, in late zygotene, than *Smc1β^−/−^* spermatocytes, the axes are not yet as fully compacted and are slightly longer than in the latest stages of *Smc1β^−/−^* spermatocytes (Suppl. Fig. S9; Fig. 4).

Quantification showed that 58% (22 of 38) of *Smc1β^−/−^Spo11^−/−^* spermatocytes carry 40 chromosome cores without non-homologous associations (Fig. 6A). The remaining 42% of cells showed more than 40 axes (Fig. 6B). None of the cells showed fewer axes. In addition to a failure to support non-homologous associations, this suggests loss of sister chromatid cohesion of some chromosomes in almost half of the cells (see below). Thus, SMC1β is required for non-homologous associations, which SMC1α alone cannot support. Other cohesins like SMC3, RAD21L and STAG3 still associate with the shortened axes indicating that the respective SMC1α-type complexes are present (Fig. 6C).

The number of DSBs depends on SPO11 dosage [69]. Would the introduction of fewer DSBs still allow homologous synapsis in the synapsis-weakened *Smc1β^−/−^* spermatocytes? *Smc1β^−/−^Spo11^+/−^* spermatocytes appeared phenotypically not different from *Smc1β^−/−^* cells, i.e. showed about the same level of asynapsis when stained for SYCP3 and SYCP1 (Fig. 6D) or pSer966-SMC1α (Fig. 6E). This indicates that wild-type levels of DSBs are not required for the level of synapsis seen in the absence of SMC1β. As expected, the number of DMC1 foci was slightly decreased in *Smc1β^−/−^Spo11^+/−^* spermatocytes compared to wt and persisted longer than in wt as described for the *Smc1β^−/−^* cells (not shown).

Further, we observed in some *Smc1β^−/−^Spo11^−/−^* spermatocytes but not in wt or single “knockout” cells what appears to be end-fusions of chromosomes, i.e. SYCP3-stained axes with a centromere signal at both ends (Fig. 6B shows 2 examples). Mostly two, but also up to 6, of the 40 or more chromosomes of about half of the cells (n = 30) were seemingly connected or at least associated at one of their ends. It appeared as if mostly the centromere-distal ends of the two chromosomes were fused.

Recently, we reported on gene dosage effects in *Smc1β^+/−^* and *Rec8^+/−^* mice [70]. A gene dosage effect of SMC1β was also observed for *Smc1β^+/−^Spo11^−/−^* spermatocytes. In a small subset of these cells (2%), no synapsis – whether homologous or non-homologous – was observed. In the remaining 98% of the cells, partial non-homologous synapsis typical for *Spo11^−/−^* spermatocytes was seen (n = 30 ; not shown).

### SMC1β is necessary for sister chromatid cohesion in prophase I

Previously, we reported a requirement for SMC1β in centromeric SCC in an okadaic acid induced metaphase I-like stage in spermatocytes and in later stages of oocyte meiosis [36], [37]. However, it remained unclear whether SMC1β is required for SCC in prophase I and whether it acts in arm and/or centromeric cohesion in early meiosis. In almost half of the *Smc1β^−/−^Spo11^−/−^* spermatocytes, such as the one shown in Figure 6B, the increased number of axes indicates loss of cohesion in some pairs of sister chromatids. However, 58% of the cells carry 40 axes, which show no signs of split sister chromatids, i.e. feature cohesion all along the chromosome arms. Thus, the remaining SMC1α complexes are sufficient for this fraction of arm cohesion at this stage. Since there is no pSer996-SMC1α in *Spo11^−/−^* cells, the non-phosphorylated form of SMC1α provides this sister chromatid cohesion. Non-phosphorylated SMC1likely acts together with non-phosphorylated SMC3, since the pSMC3 is dispersed, not associated with the chromosome axes. Thus, both SMC1α and SMC1β can contribute to arm cohesion in early prophase I.

**Figure 7:**
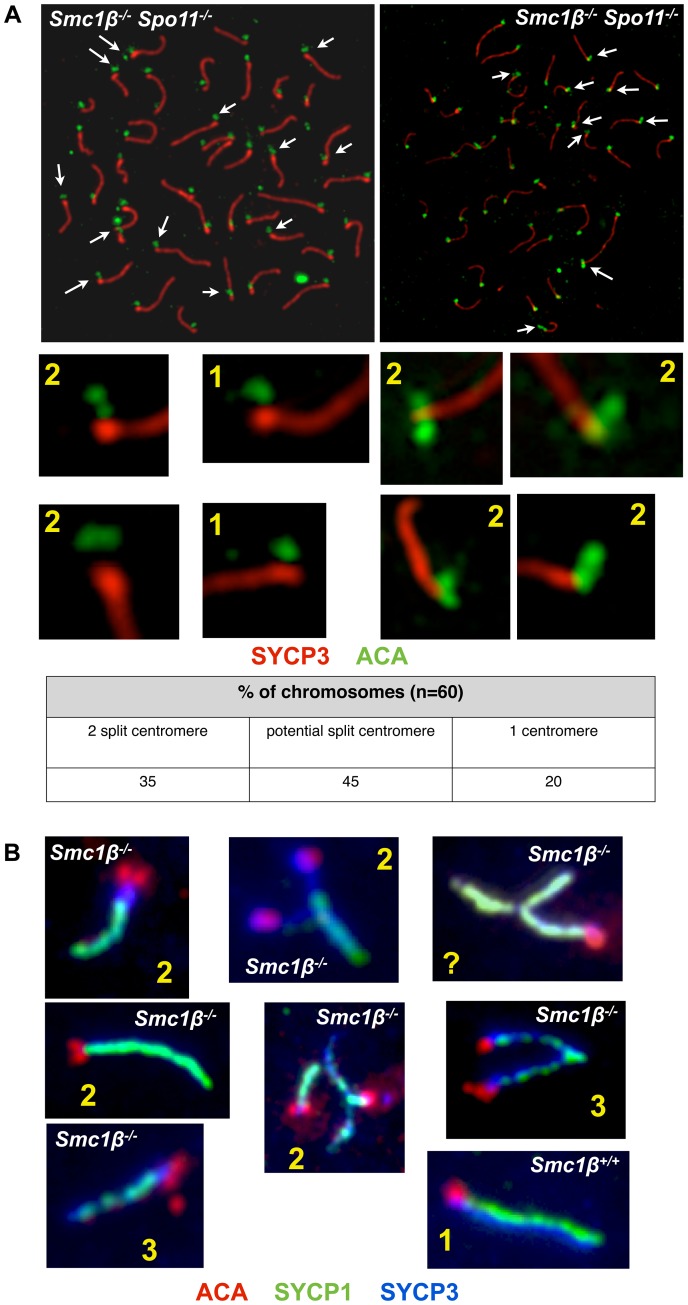
Loss of centromeric sister chromatid cohesion in *Smc1β^−/−^* and *Smc1β^−/−^Spo11^−/−^* early pachytene spermatocytes. **A** *Smc1β^−/−^Spo11^−/−^* spermatocyte chromosome spreads were stained with anti SYCP3 for AEs and anti ACA for centromeres (n = 60). The percentages of univalents showing 2 centromeres (clearly split signals), potentially split centromeres, and one centromere (non split) are provided. **B** *Smc1β^−/−^*spermatocyte chromosome spreads were stained with anti SYCP3 for AEs, SYCP1 for SCs, and anti ACA for centromeres (n = 50).

Centromeric cohesion was analyzed in *Smc1β^−/−^Spo11^−/−^* and in *Smc1β^−/−^* spermatocytes (Fig. 7). The axes of *Smc1β^−/−^Spo11^−/−^* cells were investigated for split centromere signals indicative of loss of centromeric cohesion. Clearly split centromeres were observed in 35% of the axes (n = 60 cells) (Fig. 7A). Chromosome surface spreading does not always resolve separate structures, and thus centromeres that were of large, unusual shape were distinctly quantified and found in 45% of the axes, excluding those with clearly separate centromeres. Perfectly overlapping centromeres, suggesting presumably conserved centromeric SCC, were present in the remaining 20%. This suggests a very significant contribution of SMC1β to centromeric cohesion. To confirm this, *Smc1β^−/−^* spermatocytes were similarly analyzed, although here the analysis is more complex, since the two pairs of sister chromatids with their four sister chromatids can be partially asynapsed, and may show two centromere signals because either of asynapsis or of loss of cohesion, which cannot be distinguished. We can conclude with certainty that centromeric SCC was lost only if there are three or four centromere signals. Some of the SCs showing two centromere signals may also have lost centromeric SCC. We observed 3 or 4 centromeres, i.e. loss of centromeric cohesion, in at least 3% of SCs. Another 5% of SCs showed 2 centromere signals and an unknown fraction of those may be derived from loss of SCC instead of asynapsis. Some SCs showed clearly asynapsis at the centromeric end with (30%) or without (70%) loss of centromeric cohesion. Centromeric asynapsis was as frequent as asynapsis within the chromosome arms or at the centromere-distal end. No split centromeres were observed in wt or *Spo11^−/−^* spermatocyte chromosome spreads.

## Discussion

This report addresses two major roles of cohesin in meiosis: sister chromatid cohesion and synapsis. All data and phenotypes reported here relate to these functions, either directly or indirectly. Previous reports implicated the meiosis-specific SMC1β cohesin in synapsis, post-prophase I SCC, chiasma maintenance and telomere protection [36], [37], [46]. However, important questions, which concern fundamental processes of male meiosis, remained to be resolved: (1) is SMC1β involved in SCC during prophase I of meiosis; (2) is SMC1β required for synapsis of sex chromosomes and for non-homologous synaptic associations; (3) does SMC1β affect regulation of gene expression during meiotic prophase I, and (4) is SMC1β necessary for generation and processing of meiotic, programmed DSBs? Other, more complex questions are step-wise to be resolved as new mouse models become available, such as for the distinct roles of SMC1α and SMC1β complexes in meiosis, though this study provides several insights into the functions of these two complexes. Conclusions about the role of SMC1α complexes remain indirect, since these conclusions are based on functions of SMC1α that is present in SMC1β deficient spermatocytes. Whether SMC1α fulfills the same roles in wt cells cannot be determined with certainty.

Sister chromatid cohesion as the prominent function of cohesin may be provided early in meiosis either by SMC1α- or SMC1β-based complexes. No evidence had previously been presented for a contribution of SMC1β to prophase I SCC, which did not appear to be grossly dysfunctional in *Smc1β^−/−^* prophase I cells. In addition, expression of the *Smc1β* gene starts only right after cells have entered meiosis, i.e. in early leptotene [35], [71]. Thus the previous assumption was that SMC1α complexes provide most if not all SCC before metaphase I. A contribution of SMC1β to centromeric cohesion was suggested by published data, based on experimentally driving *Smc1*®^−/−^ spermatocytes into a metaphase I-like state by okadaic acid treatment [36]. In this rather artificial set-up, 80 separate centromeres were observed in many *Smc1*®^−/−^ cells indicative of complete loss of centromeric SCC of the 4×20 mouse chromatids [36]. However, some of the cells only partially lost SCC (average of 40 centromere signals), and some showed app. 20 signals for intact centromeric SCC. We speculated that those cells suffering complete loss of SCC were of pachytene origin, while those that showed only partial loss of SCC may have originated from earlier stages. Together, it remained unclear whether SMC1β contributes to SCC earlier than metaphase I, i.e. in prophase I.

Our analyses of wt versus *Smc1β^−/−^* or *Smc1β^−/−^Spo11^−/−^* spermatocytes demonstrate an important contribution of SMC1β to zygotene/pachytene SCC. At least one-third of centromeric SCC in *Smc1β^−/−^Spo11^−/−^* spermatocytes is impaired. At least 8% of SCs in *Smc1β^−/−^* spermatocytes lose centromeric cohesion, but this number, which includes chromosomes with three centromere signals only, is probably a significant underestimation. The many centromeres that show two signals cannot be assigned to either asynaptic homologs with intact centromeric cohesion or to synapsed homologs with defective centromeric cohesion. SCC of chromosome arms appeared intact in zygotene and pachytene *Smc1β^−/−^* spermatocytes, but much higher microscopic resolution may be required to determine this. The *Smc1β^−/−^Spo11^−/−^* strain with its complete absence of homolog associations revealed in almost half of the cells more than 40 axes, mostly between 42 and 48 axes, and thus showed individual sister chromatids. Thus, about 5 to 10% of the homologs in about half of the cells lost SCC. Thus, SMC1β also contributes to arm cohesion in early prophase I. However, arm cohesion is primarily supported by SMC1α cohesin under these conditions. We did not observe partial loss of arm cohesion, which would show as bubbles, i.e. separate strands, within homologs. This “all or none” response may indicate cooperative behavior of cohesin. It shall be explored in the future why SMC1α does not provide full centromeric cohesion, and why SMC1β does relatively little for arm cohesion, i.e. how and why this functional specialization occurs. SMC1β remains associated with centromeres until metaphase II, consistent with the hypothesis that SMC1β provides the essential centromeric cohesion beyond prophase I. Formally, the synergistic effect of deficiencies in SMC1β and SPO11 renders it possible that SPO11 itself also contributes to SCC. Given the prior knowledge on SPO11 we deem this unlikely, and rather think that the much more likely interpretation is that the deficiency in SPO11 and thus absence of homologous synapsis allowed us to reveal the SPO11-independent role of SMC1β in SCC.

The analysis of *Smc1β^−/−^*, *Spo11^−/−^, and Smc1β^−/−^Spo11^−/−^* spermatocytes revealed several other important aspects of SMC1β function. It confirmed that SPO11 and thus SPO11-generated DSBs are not required for loading of cohesin proteins. The localization of RAD21 in *Spo11^−/−^* spermatocyte chromosome spreads differed from wt since much less RAD21 co-localized with the SYCP3-stained axes. More importantly, the non-homologous synaptic associations observed in *Spo11^−/−^* spermatocytes [12], [13] entirely depend on SMC1β. SMC1α alone cannot sufficiently support these associations. Thus, a second instance of functional specialization between the two SMC1 variants has been identified. Non-homologous associations might reflect an early, transient stage in the search of the homologs to find each other even before DSBs are generated. We suggest that cohesin is involved in this early stage of partner search and may provide a platform or code that allows transient, weak “trial-and-error” interactions to enhance the chance of finding the homolog.

In many *Smc1β^−/−^Spo11^−/−^* spermatocytes, but not in either single mutant or wt, several seemingly fused chromosomes were observed, where often the centromere-distal ends were fused. Earlier, we described telomere defects in *Smc1β^−/−^* meiocytes such as loss of telomeric sequences, SCs without telomeres, telomere stretches and extended bridges between telomeres consisting of stretched telomere DNA and telomeric proteins [46]. About 20 to 30% of the telomeres showed such aberrations, which we interpreted as loss of telomere protection in absence of SMC1β. The absence of protection of chromosome ends and the failure of the chromosomes to homologously synapse may contribute to end-fusions in the *Smc1β^−/−^Spo11^−/−^* spermatocytes. Homologously synapsed chromosomes are highly compacted and may have acquired a chromosome configuration prohibitive of end-fusions. This configuration does not exist in *Smc1β^−/−^Spo11^−/−^* spermatocytes. Telomere aberrations also cause failures in synapsis and may contribute to the asynapsis seen in SMC1β deficient cells.

Average chromosome length was moderately increased in *Smc1β^−/−^Spo11^−/−^* spermatocytes compared to wt cells, but variation was high. This may either reflect a slightly different developmental stage of the double-deficient cells or reduced compaction in absence of SPO11-dependent synapsis.

Why does SMC1α not bind efficiently to asynapsed, non-associated regions in *Spo11^−/−^* spermatocytes but binds to the largely unpaired sex chromosomes in wild-type cells? One may speculate that there is a specific chromatin mark or other quality in wt cells that supports SMC1α binding to the sex chromosomes.

This study further demonstrates that sex body formation depends on SMC1β and that in the absence of SMC1β, SMC1α alone cannot support PAR synapsis. The involvement of SMC1β may be direct in supporting synapsis for example through interaction of cohesin rings between the two pairs of sister chromatids. It can also be indirect if a certain axis length of the PAR is required for sufficient synapsis, and that length may not be available with the shortened *Smc1β^−/−^* axes. Our data also provides a likely explanation for the apoptosis of *Smc1β^−/−^* spermatocytes observed in mid-pachynema. While asynapsed autosomes are silenced, genes on the sex chromosome are transcriptionally activated. Thus, MSCI fails. This correlation between an MSUC reaction of autosomes and a failure of MSCI has been described and is attributed to the relocalisation of silencing factors such as γH2AX from sex chromosomes to asynaptic autosomes [9]. Made possible through the MSCI failure, the Y chromosome “spermatocyte killer gene” Zfy1/2 is expressed and probably substantially contributes to eliminating the mutant spermatocytes within the pachytene quality surveillance mechanism [26] (reviewed in [72].

Our analysis of phosphorylated forms of SMC1α and SMC3 revealed a distinct pattern of localization, particularly at the sex chromosomes. While it is difficult to entirely exclude antibody staining artifacts, the measures taken such as peptide blocking experiments, single antibody staining, secondary antibody controls, and the use of well-established anti phospho SMC antibodies, as well as the specific staining patterns like staining of the axis only of unsynapsed chromosomes (pSMC1) or staining only of the sex body chromatin (pSMC3) render artifacts unlikely. Phosphorylation of SMC1 or SMC3 may serve as a mechanism to attribute functional properties to these cohesins at different localizations. The pSer1083-SMC3 may also serve as a marker for sex bodies, and the pSer966-SMC1α as a marker for the sex chromosome axes or asynapsed axes in mutants like *Smc1β^−/−^*. Since in about 40% of *Smc1β^−/−^Spo11^−/−^* spermatocytes no pSer1083-SMC3 was detected, but all *Spo11^−/−^* spermatocytes showed pSer1083-SMC3, the phospho-form may either be unstable or these 40% of cells may have been at slightly distinct stages of spermatogenesis as their counterparts in the same mouse. The absence of pSer966-SMC1α from *Spo11^−/−^* spermatocytes indicates distinct requirements for phosphorylation of SMC1α and SMC3. The pSer966-SMC1α is observed at asynapsed chromosomes in *Smc1β^−/−^* spermatocytes but not in *Spo11^−/−^* spermatocytes that carry extensively asynapsed chromosomes. Thus, it appears as if SPO11-generated DSBs are required for SMC1α phosphorylation. SMC1α phosphorylation in somatic cells is catalyzed by ATM, whose activation in meiocytes depends on DSBs, which could explain the failure to see pSer966-SMC1α in *Spo11^−/−^* spermatocytes.

*Spo11* heterozygosity rescues the prophase I arrest of *Atm^−/−^* spermatocytes, and revealed that sex body formation and H2AX phosphorylation are supported by ATR [73], [74]. In one explanation, the reduced number of DSBs and their slower generation allows ATR to complement the ATM deficiency, particularly at zygonema and pachynema, when ATR is strongly expressed. Recent work showed, however, that ATM restricts the number of SPO11-generated DSBs and that in the absence of ATM, expression levels of SPO11 become critical [53]. Too many DSBs in an ATM-deficient, SPO11-proficient situation are deleterious and the reduced levels seen in *Atm^−/−^ Spo11^+/−^* spermatocytes are more permissive for meiotic progression. Despite this ATM-dependent DSB-triggered feedback mechanisms, the number of DSBs depends on SPO11 dosage, since *Spo11^+/−^* spermatocytes show reduced levels of DSBs [69]. Our analysis shows that wt levels of SPO11-generated DSBs are not required for SMC1β-independent non-homologous synapsis in *Smc1β^−/−^Spo11^+/−^* cells.

Several studies investigated gene expression patterns during male gametogenesis [75], [76], [77], [78]. Most of these studies compared two or more cell populations, e. g. spermatogonia with spermatocytes, spermatocytes with spermatids, or germ cells with Sertoli cells, but did not venture into analyzing meiotic sub-populations, which, for example, represent leptotene, zygotene or pachytene cells. These are very difficult to separate and to obtain in high purity and new mouse models expressing specific markers may be needed to achieve this. Therefore, juvenile male mice were used in some studies to exploit the synchrony of the first wave of meiosis during mouse puberty. However, this first wave of meiosis may not faithfully represents adult meiosis in all of its aspects. Nevertheless, altered patterns of gene expression were observed in pachytene spermatocytes compared to spermatogonia or spermatids. Some studies used mutant mouse strains such as *Spo11^−/−^* mice [79] to reveal gene expression patterns that depend on this gene and may be indicative of genes required for meiotic progression, but a clear assignment to this function requires additional analysis. In our study, we compared wt and *Smc1β^−/−^* spermatocytes at day 12 or day 16 post partum, at which spermatocytes have reached zygotene or early pachytene, respectively. FACS analysis of testis cells at day 16 revealed no major difference between wt and *Smc1β^−/−^* spermatocytes, but in *Smc1β^−/−^* samples some cells accumulated at a different position in the analysis window, indicating altered morphology. We cannot exclude a minor effect of slightly changed cellularity of the testis on the gene expression patterns. However, we assume that such an effect would be small given the absence of increased apoptosis at day 16 dpp in wt and *Smc1β^−/−^* testis, the use of littermates, and the similarity of testis tubule structure at this age. At the p-value of <0.001 more than a thousand genes differed by at least 2-fold between day 16 spermatocytes from wt and *Smc1β^−/−^* spermatocytes. A large majority (92%) of the differently regulated autosomal genes, were down-regulated. In zygonema, however, only 8 genes differed at that p-value and they were down- or up-regulated. At the least stringent p-value of <0.05, only 64 genes changed at least 2-fold, and down- and up-regulation was equally distributed among them. Therefore, a strong correlation was revealed between down-regulation of many genes in *Smc1β^−/−^* pachytene spermatocytes, and their significant asynapsis. As asynapsis is known to cause the MSUC response, we suggest that most if not all down-regulation of gene expression seen in *Smc1β^−/−^* spermatocytes is caused by asynapsis-dependent MSUC. One may also describe the down-regulation as a failure to up-regulate the genes upon synapsis in early pachynema. Provided that this view is correct, one may speculate that there may be regions on autosomes that are particularly prone to asynapsis. The presence of specific genes that are very significantly down-regulated may indicate “asynapsis^high^” regions, and thus may suggests that cohesin-dependent synapsis happens with different efficiency and perhaps timing on distinct regions of meiotic chromosomes. Our search for chromosomal clusters of aberrantly expressed genes by two different methods revealed the presence of such clusters, although only few remained significant after correcting for multiple hypothesis testing. Hence, we observed a global reduction of expression on autosomes, but we could not identify a substantial number of specific genomic regions that are particularly affected by asynapsis in *Smc1β^−/−^* pachytene cells. Genes that are not expressed before and after synapsis, very low absolute expression levels, and possible feedback mechanisms may limit the analysis and thereby may prevent the identification of putative “asynapsis^high^” regions.

Considering the few genes expressed in zygonema that are mis-regulated in *Smc1β^−/−^* spermatocytes and are either up- or down-regulated, and considering the few pachytene genes that are up-regulated in *Smc1β^−/−^* spermatocytes, we cannot exclude, however, a small direct effect of SMC1β cohesin on gene expression, possibly through interaction with transcription factors. Alternatively, these changes in gene expression may be caused by the altered loop-axis structure seen in absence of SMC1β. For example, certain chromosome regions may be aberrantly localized in chromatin loops or packed into the axis, which may affect gene expression.

Yet another phenotype of *Smc1β^−/−^* spermatocytes correlates with asynapsis: the failure to timely process DNA DSBs. While the DSBs are formed properly as indicated by analyzing SPO11-oligonucleotide complexes and by staining for RAD51 and DMC1 foci in early zygonema, these foci persist longer in *Smc1β^−/−^* spermatocytes. The repair foci are processed more slowly on asynapsed chromosomes such as sex chromosomes, and in mutants that show asynapsis. Our analysis showed that persistent repair foci localize predominantly to asynaptic chromosomes, lending support to the hypothesis that the delay in processing the repair foci is caused by asynapsis, where recombinational repair using the homolog cannot take place. The alternative pathway to repair such foci, that is the repair through recombination between sister chromatids of one homolog, is apparently not enhanced in *Smc1β^−/−^* spermatocytes, at least not to an extent that would rescue the delay in foci processing. Alternatively or additionally, more DSBs may be introduced where a chromosome or chromosomal region fails to synapse in order to further promote synapsis. The axes in the absence of SMC1β are only about half as long as those in wild-type [36]; yet the number of DSBs is only little reduced in *Smc1β^−/−^* spermatocytes. Therefore, axes length per se is unlikely to be a prime determinant of DSB numbers.

Together, this study revealed important insights into the functions of SMC1β cohesin in prophase I SCC, in autosome and sex chromosome synapsis, and it provides evidence for specific, distinct functions of SMC1β and SMC1α cohesin complexes.

Taking previous reports into account, three main functions of SMC1β have emerged that have multiple biological consequences for meiocytes: generation of the loop-axis architecture of SCs, homologous and non-homologous synapsis, as well as sister chromatid cohesion starting in early prophase I.

## Supporting Information

Figure S1 Single color images and merged color images for Figure 1A–C. **A**. Wt and *Smc1β^−/−^* spermatocyte chromosome spreads stained with anti SYCP1 for synapased chromosomes and anti HORMAD1 for unsynapsed chromosomes or chromosomal regions. **B**. Staining of the sex body in wt and *Smc1β^−/−^* spermatocyte chromosome spreads using anti SYCP1 for ASCs, anti γH2AX for the sex body or unsynapsed chromatin domains, and anti HORMAD1 for the unsynapsed sex chromosome axes or unsynapsed autosomes. **C**. Staining of the sex body in wt and *Smc1β^−/−^* spermatocyte chromosome spreads using anti SYCP3 for AEs, and anti SUMO-1 for the sex body or unsynapsed chromatin domains. **D**. Example of DAPI-staining and analysis by light micrscopy for identification of pachytene cells in the FISH experiment shown in Fig. 1D.
(TIFF)

Figure S2 Flow cytometry analysis of Hoechst 33342-stained testicular cells of mice aged 16 dpp. **A** Scheme of the distribution of spermatogenic subpopulations displays several groups of cells during the process of spermatogenesis. Cell populations are divided into spermatogonia (Spg), cells in premeiotic S phase and preleptotene (Pre-L), spermatocytes I (Spc I) and spermatocytes II (Spc II). **B–D** Total testis cells of an adult WT mouse (B) and juvenile WT and *Smc1β^−/−^* mice aged 16 dpp (C and D) were stained with Hoechst 33342 (Hoechst) and analyzed by flow cytometry. Propidium iodide allowed exclusion of dead cells (not shown). Detection of Hoechst emission using two different optical filters (Alexa Fluor 350 and Emerald 300) facilitated the distinction of cell populations of the testis. An uncharacterized population is encircled by a dashed line and likely represents zygotene/pachytene cells present in the *Smc1β^−/−^* testis. Percentages of cell populations are represented with exclusion of the smear of events in the lower left corner. Scheme in A adapted from [80].
(TIFF)

Figure S3 Spatial clustering of differentially expressed genes. Genomic domains significantly enriched for up- or down-regulated genes. Red dots indicate regions identified through conducting a two-sided Wilcoxon rank sum test inside sliding windows (window size: 10 genes). Green dots indicate regions identified through a three-state Hidden Markov Model. Significance of both types of regions was established via permutation testing resulting in False Discovery Rates (FDR). Regions with FDR<0.2 are shown for the Wilcoxon test. Dots above (below) the x-axis indicate significant clustering of up-regulated (down-regulated) genes in the respective region. Vertical black bars show fold changes of individual genes. The scale of the y-axis represents –log10(FDR) values for the genomic domains and log fold changes in case of differential gene expression. Chromosome numbers are displayed on the right side of each x-axis.
(TIFF)

Figure S4 Gene expression fold changes in testes of 16 dpp *Smc1β^−/−^* mice. Log2 transformed gene expression fold changes are displayed for all chromosomes (A) and as examples for chromosomes 1, 19 and X individually (B–D).
(TIFF)

Figure S5 Comparative expression analysis of miRNAs. Levels of the displayed mature miRNAs are at least 1.5-fold increased or decreased in the testes of at least one of three 16 dpp *Smc1β^−/−^* mice compared to wt littermates. The graph depicts miRNA ratios (n = 3, mean±s.e.m.). Dashed lines correlate with 1.5-fold increase or reduction.
(TIFF)

Figure S6 Anti pSMC3 staining control. Since the anti pSMC3 staining showed only a cloud-like pattern and not a specific axis staining as the pSMC1, we controlled for the specificity of the antibody, although both were described before, by blocking experiments using either phosphorylated epitope peptide or the non-phosphorylated peptide. Wt spermatocyte chromosome spreads were stained with anti SYCP3, anti pSMC3, and DAPI. **A** the antibodies were co-incubated with a phospho-SMC1 peptide or a phospho-SMC3 peptide. **B**. Staining of metaphase I chromosome spreads with anti SYCP3, anti pSMC3, and DAPI.
(TIF)

Figure S7 Cohesin loading does not depend on SPO11. **A**. SMC1α localization in wt and *Spo11^−/−^* spermatocytes. Spermatocyte chromosome spreads stained with anti SYCP3 for AEs and anti HORMAD1 for unsynapsed sex chromosome axes or unsynapsed autosomes and with anti SMC1α. Inset shows preferential localization of SMC1α to synapsed (HORMAD1-negative) regions. **B**. SMC3 localization in *Spo11^−/−^* spermatocytes. Spermatocyte chromosome spreads were stained with anti SYCP3 for AEs and anti HORMAD1 for unsynapsed sex chromosome axes or unsynapsed autosomes and with anti SMC3. **C**. STAG3 localization in wt and *Spo11^−/−^* spermatocytes. Spermatocyte chromosome spreads were stained with anti SYCP3 for AEs and anti STAG3. **D, E**. Localization of RAD21L in wt, *Smc1β^−/−^* and *Spo11^−/−^* spermatocytes. Spermatocyte chromosome spreads were stained with anti SYCP3 for AEs and anti HORMAD1 for unsynapsed sex chromosome axes or unsynapsed autosomes and with anti RAD21L. **F**. Localization of RAD21 in wt and *Spo11^−/−^* spermatocytes, stained as indicated. **G**. Localization of SMC1β in wt and *Spo11^−/−^* spermatocytes, stained as indicated.
(TIFF)

Figure S8 Ratio of staining intensities of either SMC1α (left, n = 12) or SMC3 (right, n = 16) on non-homologously associated (synapsed) versus non-associated (asynapsed) SYCP3-stained axes in *Spo11^−/−^* spermatocytes.
(TIFF)

Figure S9 Chromosome length in wild-type (n = 12), *Smc1β^−/−^* (n = 9) and *Smc1β^−/−^ Spo11^−/−^* (n = 8) spermatocyte spreads as measured using the ImageJ software.
(TIFF)

Figure S10 RAD51 foci in wt and *Smc1β^−/−^* spermatocytes. Spermatocyte spreads were stained with anti RAD51 and anti SYCP3. RAD51 foci were counted and the numbers of foci at different stages of meiosis is provided (n = 160).
(TIFF)

Table S1 Sequences of gene-specific primers for RT-PCR validation of microarray data.
(DOCX)

Table S2 Up- and down-regulated genes in testes of 16 dpp *Smc1β^−/−^* mice.
(DOCX)

## References

[1] Kleckner N (2006) Chiasma formation: chromatin/axis interplay and the role(s) of the synaptonemal complex. Chromosoma 115: 175–194. 16555016 10.1007/s00412-006-0055-7

[2] Costa Y, Cooke H (2007) Dissecting the mammalian synaptonemal complex using targeted mutations. Chromosome Research 15: 579. 17674147 10.1007/s10577-007-1142-1

[3] Handel MA, Schimenti JC (2010) Genetics of mammalian meiosis: regulation, dynamics and impact on fertility. Nat Rev Genet 11: 124–136. 20051984 10.1038/nrg2723

[4] Lichten M, de Massy B (2011) The impressionistic landscape of meiotic recombination. Cell 147: 267–270. 22000007 10.1016/j.cell.2011.09.038 PMC3263351

[5] Youds JL, Boulton SJ (2011) The choice in meiosis - defining the factors that influence crossover or non-crossover formation. J Cell Sci 124: 501–513. 21282472 10.1242/jcs.074427

[6] Sasaki M, Lange J, Keeney S (2010) Genome destabilization by homologous recombination in the germ line. Nat Rev Mol Cell Biol 11: 182–195. 20164840 10.1038/nrm2849 PMC3073813

[7] Keeney S (2008) Spo11 and the Formation of DNA Double-Strand Breaks in Meiosis. Genome Dyn Stab 2: 81–123. 21927624 10.1007/7050_2007_026 PMC3172816

[8] Yanowitz J (2010) Meiosis: making a break for it. Curr Opin Cell Biol 22: 744–751. 20829015 10.1016/j.ceb.2010.08.016 PMC3003294

[9] Burgoyne PS, Mahadevaiah SK, Turner JM (2009) The consequences of asynapsis for mammalian meiosis. Nat Rev Genet 10: 207–216. 19188923 10.1038/nrg2505

[10] Fukuda T, Daniel K, Wojtasz L, Toth A, Hoog C (2010) A novel mammalian HORMA domain-containing protein, HORMAD1, preferentially associates with unsynapsed meiotic chromosomes. Exp Cell Res 316: 158–171. 19686734 10.1016/j.yexcr.2009.08.007

[11] Wojtasz L, Daniel K, Roig I, Bolcun-Filas E, Xu H, et al (2009) Mouse HORMAD1 and HORMAD2, two conserved meiotic chromosomal proteins, are depleted from synapsed chromosome axes with the help of TRIP13 AAA-ATPase. PLoS Genet 5: e1000702. 19851446 10.1371/journal.pgen.1000702 PMC2758600

[12] Romanienko PJ, Camerini-Otero RD (2000) The mouse Spo11 gene is required for meiotic chromosome synapsis. Mol Cell 6: 975–987. 11106738 10.1016/s1097-2765(00)00097-6

[13] Baudat F, Manova K, Yuen JP, Jasin M, Keeney S (2000) Chromosome synapsis defects and sexually dimorphic meiotic progression in mice lacking Spo11. Mol Cell 6: 989–998. 11106739 10.1016/s1097-2765(00)00098-8

[14] Ellis N, Goodfellow PN (1989) The mammalian pseudoautosomal region. Trends Genet 5: 406–410. 2696184 10.1016/0168-9525(89)90199-6

[15] Perry J, Palmer S, Gabriel A, Ashworth A (2001) A short pseudoautosomal region in laboratory mice. Genome Res 11: 1826–1832. 11691846 10.1101/gr.203001 PMC311143

[16] Anderson LK, Reeves A, Webb LM, Ashley T (1999) Distribution of crossing over on mouse synaptonemal complexes using immunofluorescent localization of MLH1 protein. Genetics 151: 1569–1579. 10101178 10.1093/genetics/151.4.1569 PMC1460565

[17] Kauppi L, Barchi M, Baudat F, Romanienko PJ, Keeney S, et al (2011) Distinct properties of the XY pseudoautosomal region crucial for male meiosis. Science 331: 916–920. 21330546 10.1126/science.1195774 PMC3151169

[18] Inagaki A, Schoenmakers S, Baarends WM (2010) DNA double strand break repair, chromosome synapsis and transcriptional silencing in meiosis. Epigenetics 5: 255–266. 20364103 10.4161/epi.5.4.11518

[19] Yan W, McCarrey JR (2009) Sex chromosome inactivation in the male. Epigenetics 4: 452–456. 19838052 10.4161/epi.4.7.9923 PMC3052906

[20] Handel MA (2004) The XY body: a specialized meiotic chromatin domain. Exp Cell Res 296: 57–63. 15120994 10.1016/j.yexcr.2004.03.008

[21] Fernandez-Capetillo O, Mahadevaiah SK, Celeste A, Romanienko PJ, Camerini-Otero RD, et al (2003) H2AX is required for chromatin remodeling and inactivation of sex chromosomes in male mouse meiosis. Dev Cell 4: 497–508. 12689589 10.1016/s1534-5807(03)00093-5

[22] Page J, de la Fuente R, Manterola M, Parra MT, Viera A, et al (2012) Inactivation or non-reactivation: what accounts better for the silence of sex chromosomes during mammalian male meiosis? Chromosoma 121: 307–326. 22366883 10.1007/s00412-012-0364-y

[23] Turner JM, Mahadevaiah SK, Fernandez-Capetillo O, Nussenzweig A, Xu X, et al (2005) Silencing of unsynapsed meiotic chromosomes in the mouse. Nat Genet 37: 41–47. 15580272 10.1038/ng1484

[24] Mahadevaiah SK, Bourc'his D, de Rooij DG, Bestor TH, Turner JM, et al (2008) Extensive meiotic asynapsis in mice antagonises meiotic silencing of unsynapsed chromatin and consequently disrupts meiotic sex chromosome inactivation. J Cell Biol 182: 263–276. 18663141 10.1083/jcb.200710195 PMC2483523

[25] Turner JM (2007) Meiotic sex chromosome inactivation. Development 134: 1823–1831. 17329371 10.1242/dev.000018

[26] Royo H, Polikiewicz G, Mahadevaiah SK, Prosser H, Mitchell M, et al (2010) Evidence that meiotic sex chromosome inactivation is essential for male fertility. Curr Biol 20: 2117–2123. 21093264 10.1016/j.cub.2010.11.010

[27] Nasmyth K, Haering CH (2009) Cohesin: its roles and mechanisms. Annu Rev Genet 43: 525–558. 19886810 10.1146/annurev-genet-102108-134233

[28] Shintomi K, Hirano T (2010) Sister chromatid resolution: a cohesin releasing network and beyond. Chromosoma 119: 459–467. 20352243 10.1007/s00412-010-0271-z

[29] Onn I, Heidinger-Pauli JM, Guacci V, Unal E, Koshland DE (2008) Sister chromatid cohesion: a simple concept with a complex reality. Annu Rev Cell Dev Biol 24: 105–129. 18616427 10.1146/annurev.cellbio.24.110707.175350

[30] Wood AJ, Severson AF, Meyer BJ (2010) Condensin and cohesin complexity: the expanding repertoire of functions. Nat Rev Genet 11: 391–404. 20442714 10.1038/nrg2794 PMC3491780

[31] Nasmyth K (2011) Cohesin: a catenase with separate entry and exit gates? Nat Cell Biol 13: 1170–1177. 21968990 10.1038/ncb2349

[32] Haering CH, Jessberger R (2012) Cohesin in determining chromosome architecture. Exp Cell Res 318: 1386–1393. 22472347 10.1016/j.yexcr.2012.03.016

[33] Uhlmann F (2011) Cohesin subunit Rad21L, the new kid on the block has new ideas. EMBO Rep 12: 183–184. 21311562 10.1038/embor.2011.24 PMC3059923

[34] Jessberger R (2011) Cohesin complexes get more complex: the novel kleisin RAD21L. Cell Cycle 10: 2053–2054. 21597328 10.4161/cc.10.13.15802

[35] Revenkova E, Eijpe M, Heyting C, Gross B, Jessberger R (2001) Novel meiosis-specific isoform of mammalian SMC1. Mol Cell Biol 21: 6984–6998. 11564881 10.1128/MCB.21.20.6984-6998.2001 PMC99874

[36] Revenkova E, Eijpe M, Heyting C, Hodges CA, Hunt PA, et al (2004) Cohesin SMC1 beta is required for meiotic chromosome dynamics, sister chromatid cohesion and DNA recombination. Nat Cell Biol 6: 555–562. 15146193 10.1038/ncb1135

[37] Hodges CA, Revenkova E, Jessberger R, Hassold TJ, Hunt PA (2005) SMC1beta-deficient female mice provide evidence that cohesins are a missing link in age-related nondisjunction. Nat Genet 37: 1351–1355. 16258540 10.1038/ng1672

[38] Strom L, Sjogren C (2007) Chromosome segregation and double-strand break repair - a complex connection. Curr Opin Cell Biol 19: 344–349. 17466504 10.1016/j.ceb.2007.04.003

[39] Watrin E, Peters JM (2006) Cohesin and DNA damage repair. Exp Cell Res 312: 2687–2693. 16876157 10.1016/j.yexcr.2006.06.024

[40] Feeney KM, Wasson CW, Parish JL (2010) Cohesin: a regulator of genome integrity and gene expression. Biochem J 428: 147–161. 20462401 10.1042/BJ20100151

[41] Cortes-Ledesma F, de Piccoli G, Haber JE, Aragon L, Aguilera A (2007) SMC proteins, new players in the maintenance of genomic stability. Cell Cycle 6: 914–918. 17404505 10.4161/cc.6.8.4107

[42] Wendt KS, Peters JM (2009) How cohesin and CTCF cooperate in regulating gene expression. Chromosome Res 17: 201–214. 19308701 10.1007/s10577-008-9017-7

[43] Merkenschlager M (2010) Cohesin: a global player in chromosome biology with local ties to gene regulation. Curr Opin Genet Dev 20: 555–561. 20541931 10.1016/j.gde.2010.05.007

[44] Dorsett D (2011) Cohesin: genomic insights into controlling gene transcription and development. Curr Opin Genet Dev 21: 199–206. 21324671 10.1016/j.gde.2011.01.018 PMC3070859

[45] Bose T, Gerton JL (2010) Cohesinopathies, gene expression, and chromatin organization. J Cell Biol 189: 201–210. 20404106 10.1083/jcb.200912129 PMC2856913

[46] Adelfalk C, Janschek J, Revenkova E, Blei C, Liebe B, et al (2009) Cohesin SMC1beta protects telomeres in meiocytes. J Cell Biol 187: 185–199. 19841137 10.1083/jcb.200808016 PMC2768837

[47] Scherthan H, Jerratsch M, Li B, Smith S, Hulten M, et al (2000) Mammalian meiotic telomeres: protein composition and redistribution in relation to nuclear pores. Mol Biol Cell 11: 4189–4203. 11102517 10.1091/mbc.11.12.4189 PMC15066

[48] Peters AH, Plug AW, van Vugt MJ, de Boer P (1997) A drying-down technique for the spreading of mammalian meiocytes from the male and female germline. Chromosome Res 5: 66–68. 9088645 10.1023/a:1018445520117

[49] Lee J, Hirano T (2011) RAD21L, a novel cohesin subunit implicated in linking homologous chromosomes in mammalian meiosis. J Cell Biol 192: 263–276. 21242291 10.1083/jcb.201008005 PMC3172173

[50] Eijpe M, Heyting C, Gross B, Jessberger R (2000) Association of mammalian SMC1 and SMC3 proteins with meiotic chromosomes and synaptonemal complexes. J Cell Sci 113 Pt 4: 673–682. 10652260 10.1242/jcs.113.4.673

[51] Takada Y, Naruse C, Costa Y, Shirakawa T, Tachibana M, et al (2011) HP1gamma links histone methylation marks to meiotic synapsis in mice. Development 138: 4207–4217. 21896631 10.1242/dev.064444

[52] Baumann C, Daly CM, McDonnell SM, Viveiros MM, De La Fuente R (2011) Chromatin configuration and epigenetic landscape at the sex chromosome bivalent during equine spermatogenesis. Chromosoma 120: 227–244. 21274552 10.1007/s00412-010-0306-5 PMC3100478

[53] Lange J, Pan J, Cole F, Thelen MP, Jasin M, et al (2011) ATM controls meiotic double-strand-break formation. Nature 479: 237–240. 22002603 10.1038/nature10508 PMC3213282

[54] Rozen S, Skaletsky H (2000) Primer3 on the WWW for general users and for biologist programmers. Methods Mol Biol 132: 365–386. 10547847 10.1385/1-59259-192-2:365

[55] Wilcoxon F (1945) Individual comparisons by ranking methods. Biometrics Bulletin 1: 80–83.

[56] Seifert M, Strickert M, Schliep A, Grosse I (2011) Exploiting prior knowledge and gene distances in the analysis of tumor expression profiles with extended Hidden Markov Models. Bioinformatics 27: 1645–1652. 21511716 10.1093/bioinformatics/btr199

[57] Novak I, Wang H, Revenkova E, Jessberger R, Scherthan H, et al (2008) Cohesin Smc1beta determines meiotic chromatin axis loop organization. J Cell Biol 180: 83–90. 18180366 10.1083/jcb.200706136 PMC2213612

[58] de Carvalho CE, Colaiacovo MP (2006) SUMO-mediated regulation of synaptonemal complex formation during meiosis. Genes Dev 20: 1986–1992. 16882975 10.1101/gad.1457806

[59] Rogers RS, Inselman A, Handel MA, Matunis MJ (2004) SUMO modified proteins localize to the XY body of pachytene spermatocytes. Chromosoma 113: 233–243. 15349788 10.1007/s00412-004-0311-7

[60] Vigodner M, Morris PL (2005) Testicular expression of small ubiquitin-related modifier-1 (SUMO-1) supports multiple roles in spermatogenesis: silencing of sex chromosomes in spermatocytes, spermatid microtubule nucleation, and nuclear reshaping. Dev Biol 282: 480–492. 15950612 10.1016/j.ydbio.2005.03.034

[61] Fukuda T, Pratto F, Schimenti JC, Turner JM, Camerini-Otero RD, et al (2012) Phosphorylation of Chromosome Core Components May Serve as Axis Marks for the Status of Chromosomal Events during Mammalian Meiosis. PLoS Genet 8: e1002485. 22346761 10.1371/journal.pgen.1002485 PMC3276554

[62] Yazdi PT, Wang Y, Zhao S, Patel N, Lee EY, et al (2002) SMC1 is a downstream effector in the ATM/NBS1 branch of the human S-phase checkpoint. Genes Dev 16: 571–582. 11877377 10.1101/gad.970702 PMC155356

[63] Kim ST, Xu B, Kastan MB (2002) Involvement of the cohesin protein, Smc1, in Atm-dependent and independent responses to DNA damage. Genes Dev 16: 560–570. 11877376 10.1101/gad.970602 PMC155347

[64] Liefshitz B, Kupiec M (2011) Roles of RSC, Rad59, and cohesin in double-strand break repair. Mol Cell Biol 31: 3921–3923. 21844225 10.1128/MCB.05974-11 PMC3187367

[65] Heidinger-Pauli JM, Unal E, Guacci V, Koshland D (2008) The kleisin subunit of cohesin dictates damage-induced cohesion. Mol Cell 31: 47–56. 18614046 10.1016/j.molcel.2008.06.005

[66] James RD, Schmiesing JA, Peters AH, Yokomori K, Disteche CM (2002) Differential association of SMC1alpha and SMC3 proteins with meiotic chromosomes in wild-type and SPO11-deficient male mice. Chromosome Res 10: 549–560. 12498344 10.1023/a:1020910601858

[67] Neale MJ, Pan J, Keeney S (2005) Endonucleolytic processing of covalent protein-linked DNA double-strand breaks. Nature 436: 1053–1057. 16107854 10.1038/nature03872 PMC1262668

[68] Bellani MA, Boateng KA, McLeod D, Camerini-Otero RD (2010) The expression profile of the major mouse SPO11 isoforms indicates that SPO11beta introduces double strand breaks and suggests that SPO11alpha has an additional role in prophase in both spermatocytes and oocytes. Mol Cell Biol 30: 4391–4403. 20647542 10.1128/MCB.00002-10 PMC2937527

[69] Cole F, Kauppi L, Lange J, Roig I, Wang R, et al (2012) Homeostatic control of recombination is implemented progressively in mouse meiosis. Nat Cell Biol 14: 424–430. 22388890 10.1038/ncb2451 PMC3319518

[70] Murdoch B, Owen N, Stevense M, Smith H, Nagaoka S, et al (2013) Altered cohesin gene dosage affects Mammalian meiotic chromosome structure and behavior. PLoS Genet 9: e1003241. 23408896 10.1371/journal.pgen.1003241 PMC3567145

[71] Eijpe M, Offenberg H, Jessberger R, Revenkova E, Heyting C (2003) Meiotic cohesin REC8 marks the axial elements of rat synaptonemal complexes before cohesins SMC1beta and SMC3. J Cell Biol 160: 657–670. 12615909 10.1083/jcb.200212080 PMC2173354

[72] Toth A, Jessberger R (2010) Male meiosis: Y keep it silenced? Curr Biol 20: R1022–1024. 21145018 10.1016/j.cub.2010.11.002

[73] Bellani MA, Romanienko PJ, Cairatti DA, Camerini-Otero RD (2005) SPO11 is required for sex-body formation, and Spo11 heterozygosity rescues the prophase arrest of Atm-/- spermatocytes. J Cell Sci 118: 3233–3245. 15998665 10.1242/jcs.02466

[74] Barchi M, Roig I, Di Giacomo M, de Rooij DG, Keeney S, et al (2008) ATM promotes the obligate XY crossover and both crossover control and chromosome axis integrity on autosomes. PLoS Genet 4: e1000076. 18497861 10.1371/journal.pgen.1000076 PMC2374915

[75] Almstrup K, Nielsen JE, Hansen MA, Tanaka M, Skakkebaek NE, et al (2004) Analysis of cell-type-specific gene expression during mouse spermatogenesis. Biol Reprod 70: 1751–1761. 14960480 10.1095/biolreprod.103.026575

[76] Rossi P, Dolci S, Sette C, Capolunghi F, Pellegrini M, et al (2004) Analysis of the gene expression profile of mouse male meiotic germ cells. Gene Expr Patterns 4: 267–281. 15053975 10.1016/j.modgep.2003.11.003

[77] Chalmel F, Rolland AD, Niederhauser-Wiederkehr C, Chung SS, Demougin P, et al (2007) The conserved transcriptome in human and rodent male gametogenesis. Proc Natl Acad Sci U S A 104: 8346–8351. 17483452 10.1073/pnas.0701883104 PMC1864911

[78] Pang AL, Johnson W, Ravindranath N, Dym M, Rennert OM, et al (2006) Expression profiling of purified male germ cells: stage-specific expression patterns related to meiosis and postmeiotic development. Physiol Genomics 24: 75–85. 16291737 10.1152/physiolgenomics.00215.2004

[79] Smirnova NA, Romanienko PJ, Khil PP, Camerini-Otero RD (2006) Gene expression profiles of Spo11-/- mouse testes with spermatocytes arrested in meiotic prophase I. Reproduction 132: 67–77. 16816334 10.1530/rep.1.00997

[80] Bastos H, Lassalle B, Chicheportiche A, Riou L, Testart J, et al (2005) Flow cytometric characterization of viable meiotic and postmeiotic cells by Hoechst 33342 in mouse spermatogenesis. Cytometry A 65: 40–49. 15779065 10.1002/cyto.a.20129

