# Supplementary figures and images for: Meiotic Cohesin SMC1β Provides Prophase I Centromeric Cohesion and Is Required for Multiple Synapsis-Associated Functions
Source: PLoS Genet. 2013 Dec 26;9(12):e1003985. doi: 10.1371/journal.pgen.1003985 (PMC3873225; doi:10.1371/journal.pgen.1003985)

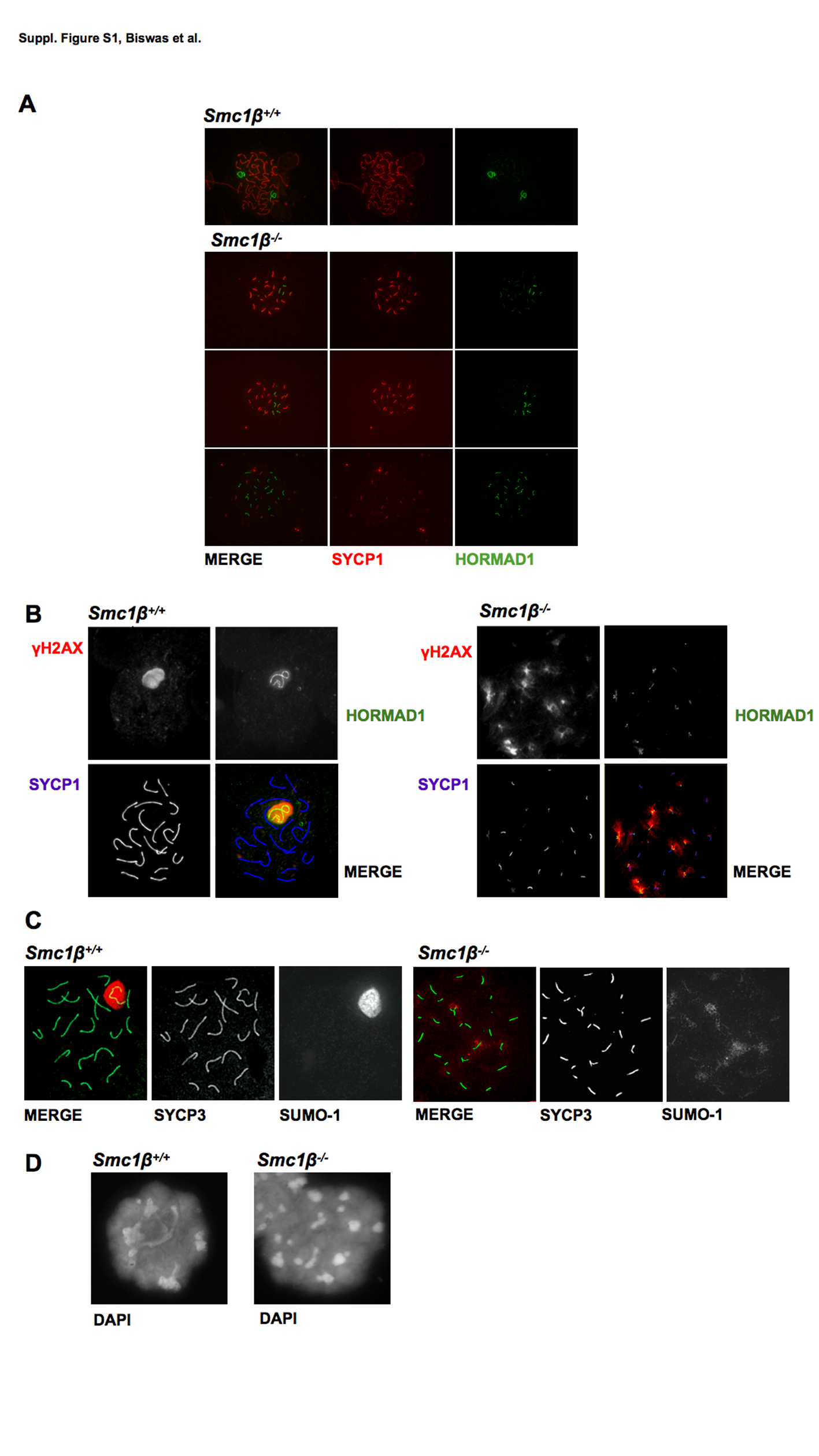

Supplement: Figure S1 — Single color images and merged color images for Figure 1A–C. A. Wt and Smc1β−/− spermatocyte chromosome spreads stained with anti SYCP1 for synapased chromosomes and anti HORMAD1 for unsynapsed chromosomes or chromosomal regions. B. Staining of the sex body in wt and Smc1β−/− spermatocyte chromosome spreads using anti SYCP1 for ASCs, anti γH2AX for the sex body or unsynapsed chromatin domains, and anti HORMAD1 for the unsynapsed sex chromosome axes or unsynapsed autosomes. C. Staining of the sex body in wt and Smc1β−/− spermatocyte chromosome spreads using anti SYCP3 for AEs, and anti SUMO-1 for the sex body or unsynapsed chromatin domains. D. Example of DAPI-staining and analysis by light micrscopy for identification of pachytene cells in the FISH experiment shown in Fig. 1D. (TIFF) [file pgen.1003985.s001.tiff]

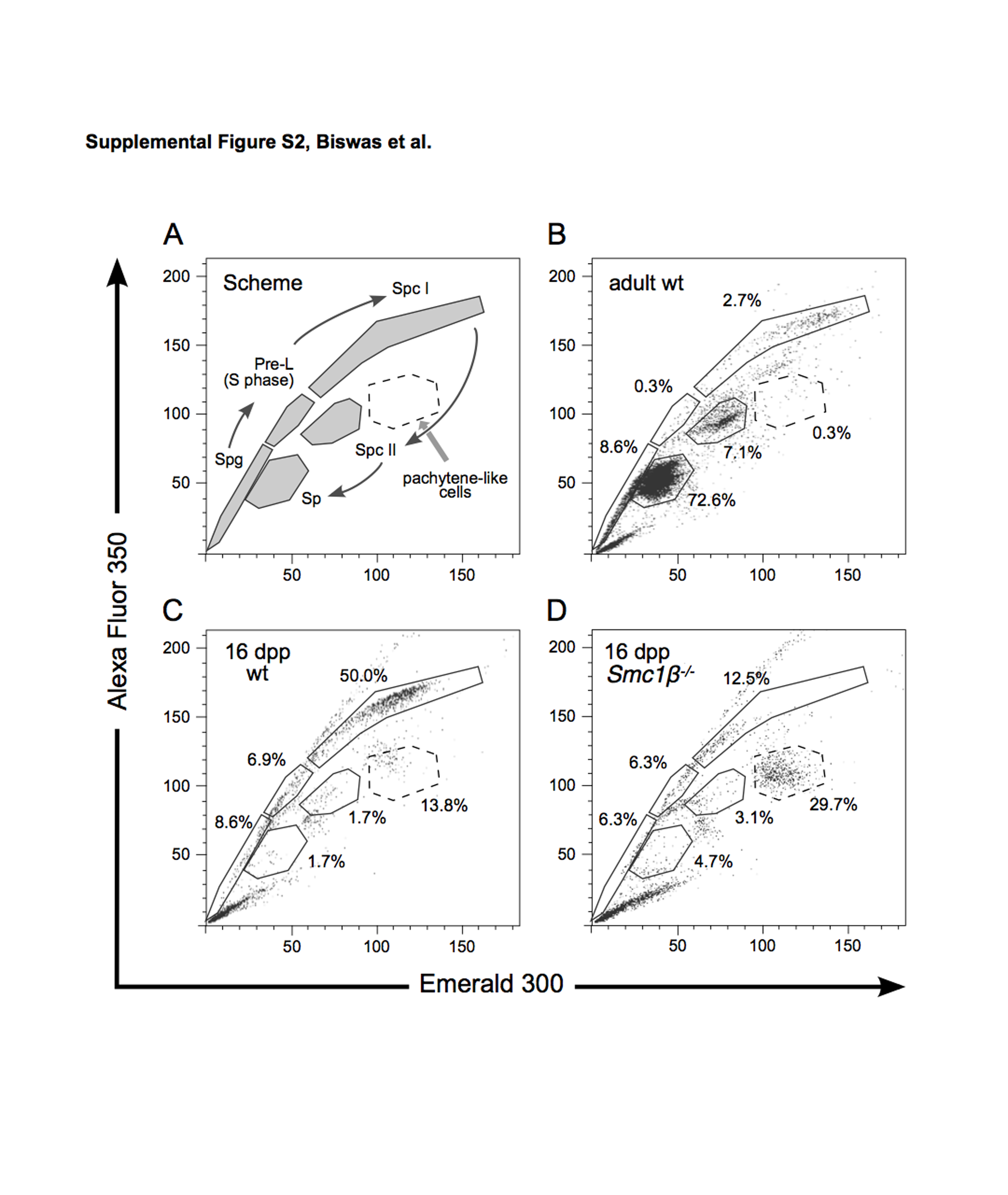

Supplement: Figure S2 — Flow cytometry analysis of Hoechst 33342-stained testicular cells of mice aged 16 dpp. A Scheme of the distribution of spermatogenic subpopulations displays several groups of cells during the process of spermatogenesis. Cell populations are divided into spermatogonia (Spg), cells in premeiotic S phase and preleptotene (Pre-L), spermatocytes I (Spc I) and spermatocytes II (Spc II). B–D Total testis cells of an adult WT mouse (B) and juvenile WT and Smc1β−/− mice aged 16 dpp (C and D) were stained with Hoechst 33342 (Hoechst) and analyzed by flow cytometry. Propidium iodide allowed exclusion of dead cells (not shown). Detection of Hoechst emission using two different optical filters (Alexa Fluor 350 and Emerald 300) facilitated the distinction of cell populations of the testis. An uncharacterized population is encircled by a dashed line and likely represents zygotene/pachytene cells present in the Smc1β−/− testis. Percentages of cell populations are represented with exclusion of the smear of events in the lower left corner. Scheme in A adapted from [80]. (TIFF) [file pgen.1003985.s002.tiff]

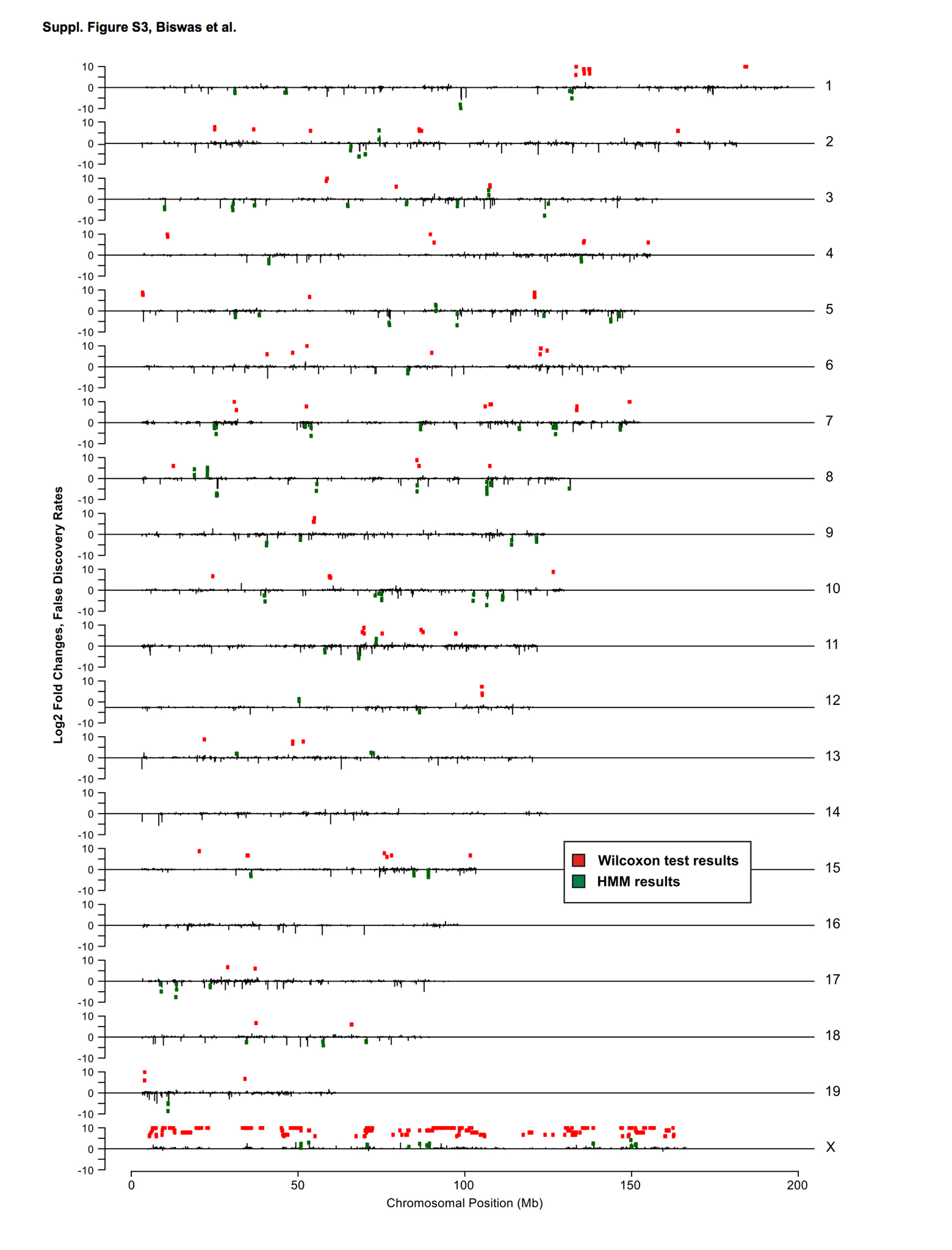

Supplement: Figure S3 — Spatial clustering of differentially expressed genes. Genomic domains significantly enriched for up- or down-regulated genes. Red dots indicate regions identified through conducting a two-sided Wilcoxon rank sum test inside sliding windows (window size: 10 genes). Green dots indicate regions identified through a three-state Hidden Markov Model. Significance of both types of regions was established via permutation testing resulting in False Discovery Rates (FDR). Regions with FDR<0.2 are shown for the Wilcoxon test. Dots above (below) the x-axis indicate significant clustering of up-regulated (down-regulated) genes in the respective region. Vertical black bars show fold changes of individual genes. The scale of the y-axis represents –log10(FDR) values for the genomic domains and log fold changes in case of differential gene expression. Chromosome numbers are displayed on the right side of each x-axis. (TIFF) [file pgen.1003985.s003.tiff]

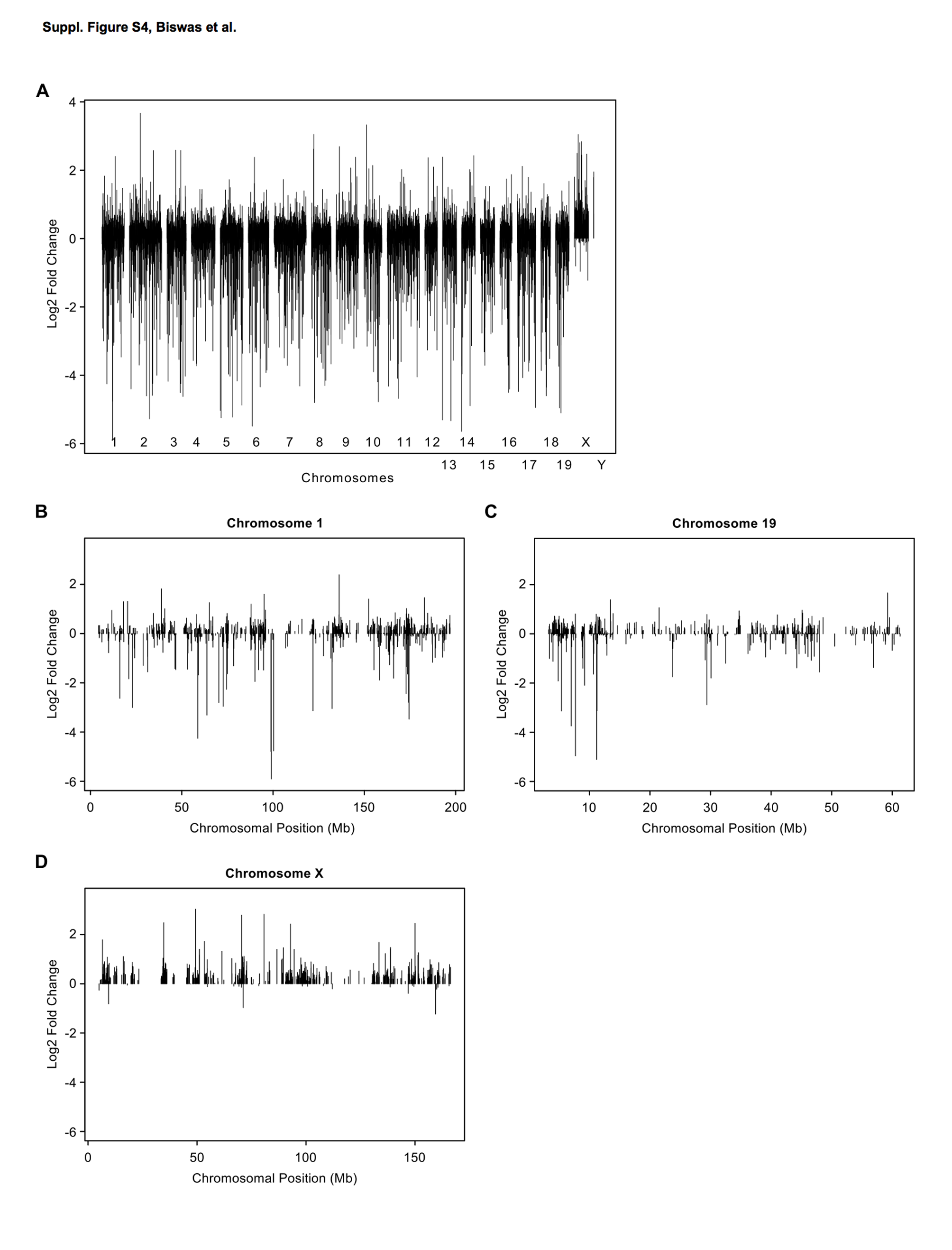

Supplement: Figure S4 — Gene expression fold changes in testes of 16 dpp Smc1β−/− mice. Log2 transformed gene expression fold changes are displayed for all chromosomes (A) and as examples for chromosomes 1, 19 and X individually (B–D). (TIFF) [file pgen.1003985.s004.tiff]

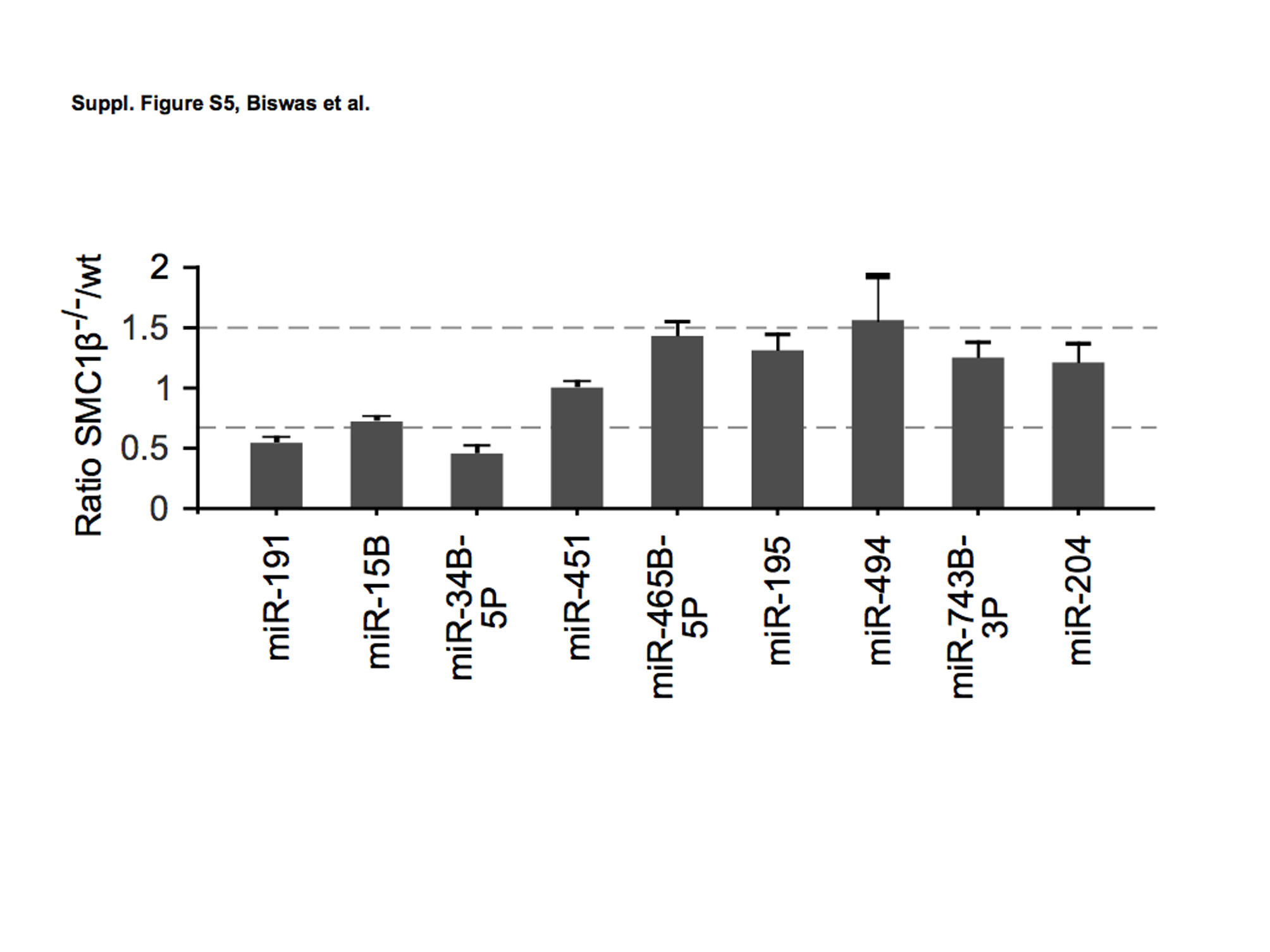

Supplement: Figure S5 — Comparative expression analysis of miRNAs. Levels of the displayed mature miRNAs are at least 1.5-fold increased or decreased in the testes of at least one of three 16 dpp Smc1β−/− mice compared to wt littermates. The graph depicts miRNA ratios (n = 3, mean±s.e.m.). Dashed lines correlate with 1.5-fold increase or reduction. (TIFF) [file pgen.1003985.s005.tiff]

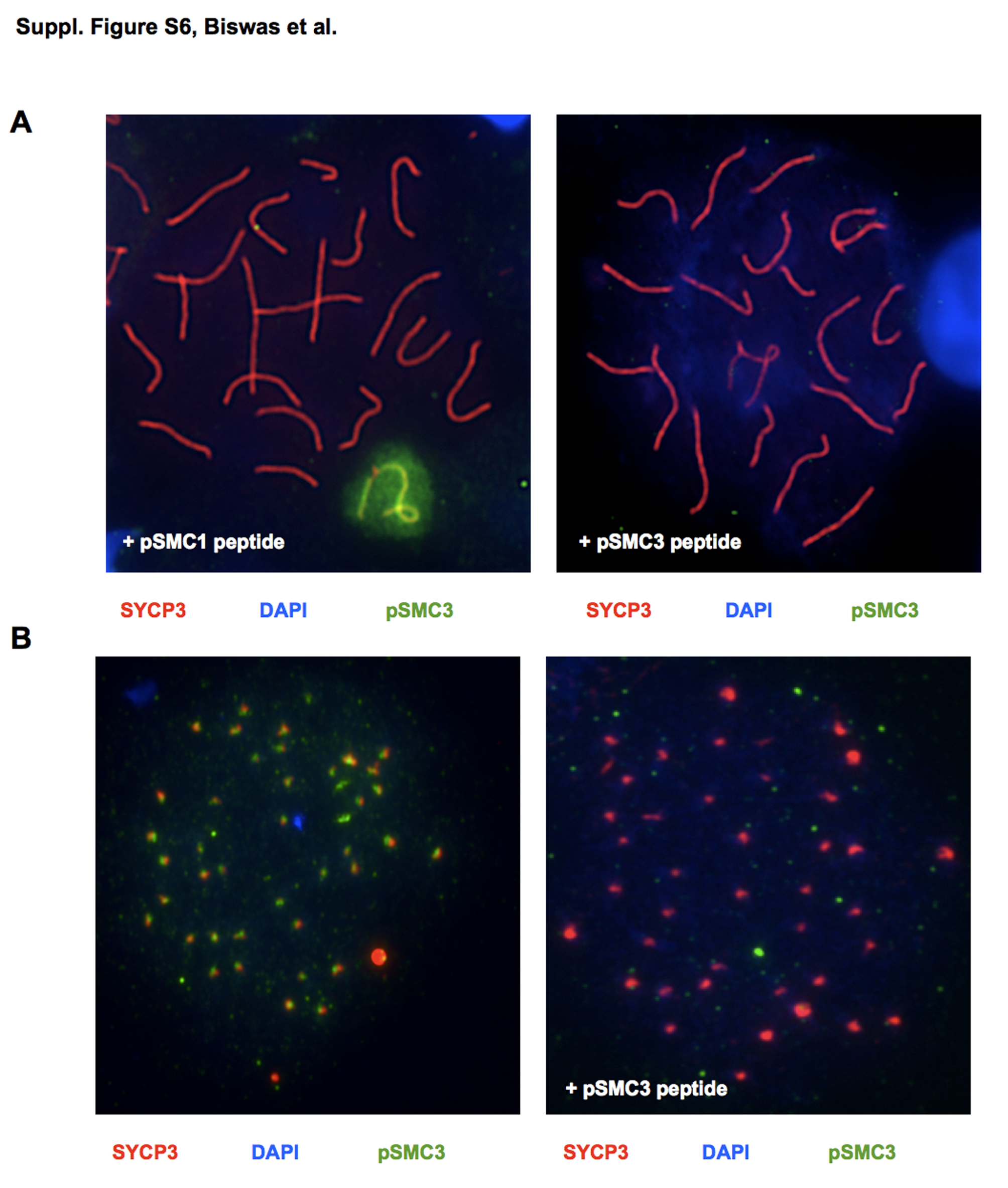

Supplement: Figure S6 — Anti pSMC3 staining control. Since the anti pSMC3 staining showed only a cloud-like pattern and not a specific axis staining as the pSMC1, we controlled for the specificity of the antibody, although both were described before, by blocking experiments using either phosphorylated epitope peptide or the non-phosphorylated peptide. Wt spermatocyte chromosome spreads were stained with anti SYCP3, anti pSMC3, and DAPI. A the antibodies were co-incubated with a phospho-SMC1 peptide or a phospho-SMC3 peptide. B. Staining of metaphase I chromosome spreads with anti SYCP3, anti pSMC3, and DAPI. (TIF) [file pgen.1003985.s006.tif]

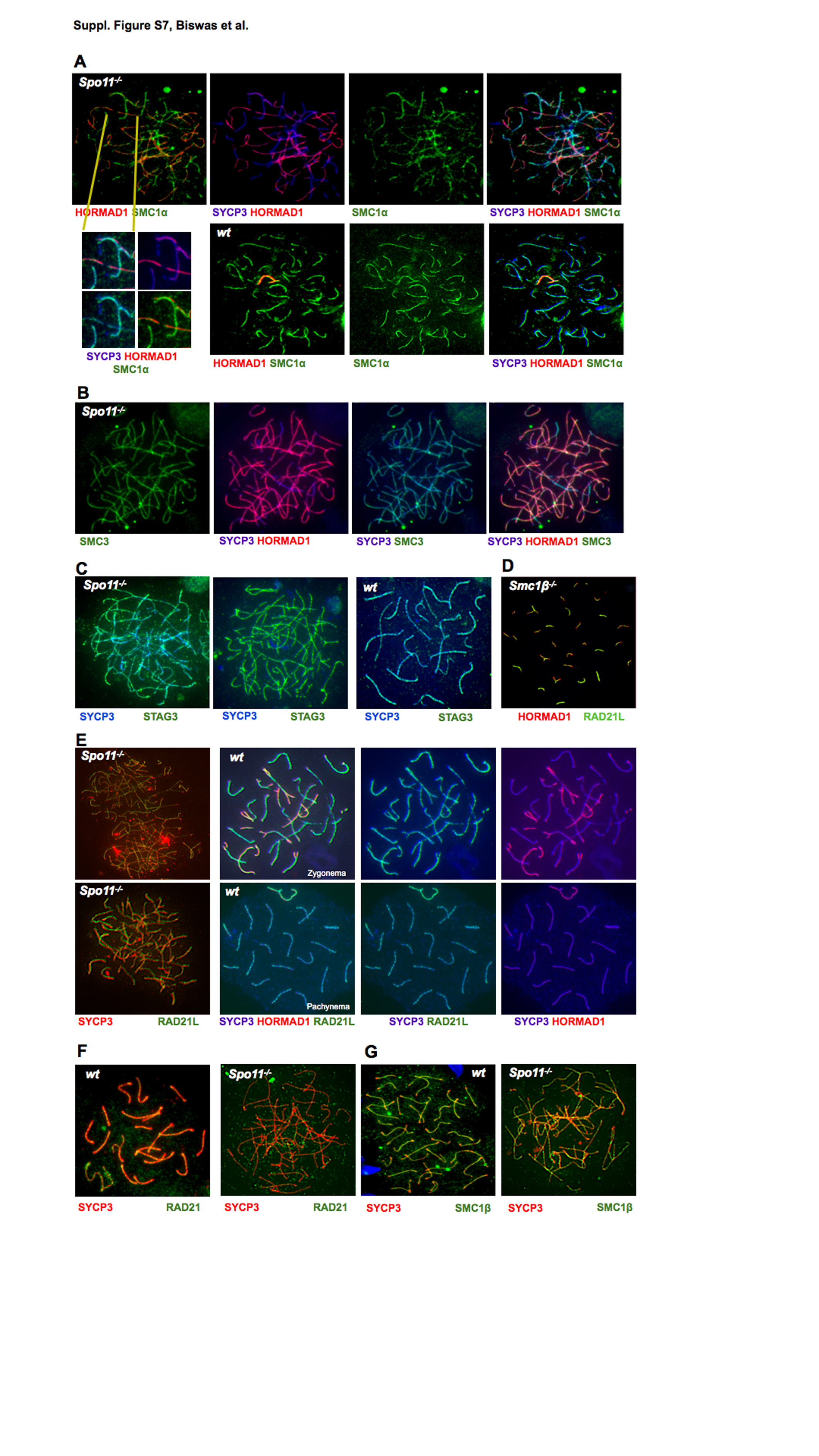

Supplement: Figure S7 — Cohesin loading does not depend on SPO11. A. SMC1α localization in wt and Spo11−/− spermatocytes. Spermatocyte chromosome spreads stained with anti SYCP3 for AEs and anti HORMAD1 for unsynapsed sex chromosome axes or unsynapsed autosomes and with anti SMC1α. Inset shows preferential localization of SMC1α to synapsed (HORMAD1-negative) regions. B. SMC3 localization in Spo11−/− spermatocytes. Spermatocyte chromosome spreads were stained with anti SYCP3 for AEs and anti HORMAD1 for unsynapsed sex chromosome axes or unsynapsed autosomes and with anti SMC3. C. STAG3 localization in wt and Spo11−/− spermatocytes. Spermatocyte chromosome spreads were stained with anti SYCP3 for AEs and anti STAG3. D, E. Localization of RAD21L in wt, Smc1β−/− and Spo11−/− spermatocytes. Spermatocyte chromosome spreads were stained with anti SYCP3 for AEs and anti HORMAD1 for unsynapsed sex chromosome axes or unsynapsed autosomes and with anti RAD21L. F. Localization of RAD21 in wt and Spo11−/− spermatocytes, stained as indicated. G. Localization of SMC1β in wt and Spo11−/− spermatocytes, stained as indicated. (TIFF) [file pgen.1003985.s007.tiff]

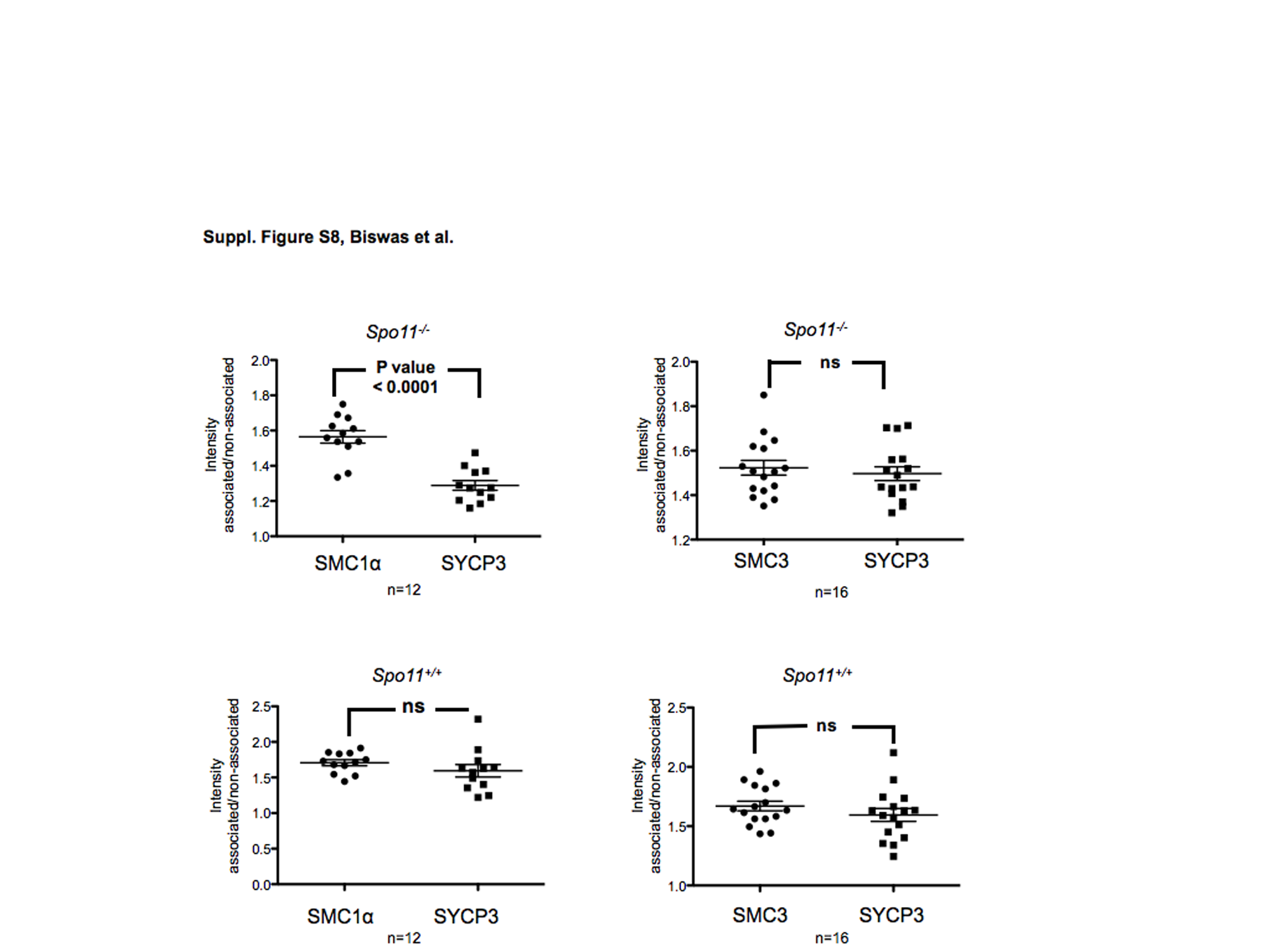

Supplement: Figure S8 — Ratio of staining intensities of either SMC1α (left, n = 12) or SMC3 (right, n = 16) on non-homologously associated (synapsed) versus non-associated (asynapsed) SYCP3-stained axes in Spo11−/− spermatocytes. (TIFF) [file pgen.1003985.s008.tiff]

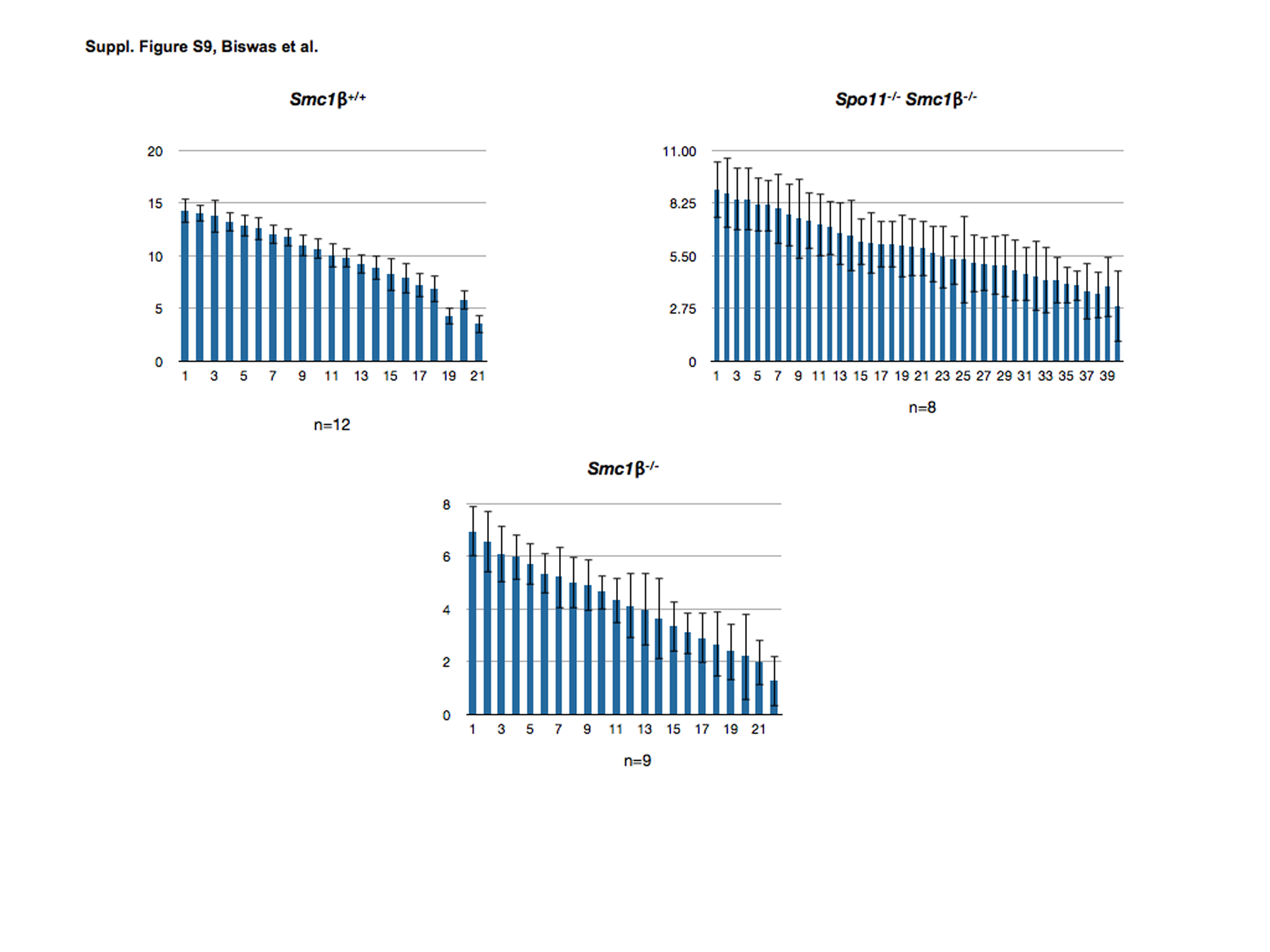

Supplement: Figure S9 — Chromosome length in wild-type (n = 12), Smc1β−/− (n = 9) and Smc1β−/− Spo11−/− (n = 8) spermatocyte spreads as measured using the ImageJ software. (TIFF) [file pgen.1003985.s009.tiff]

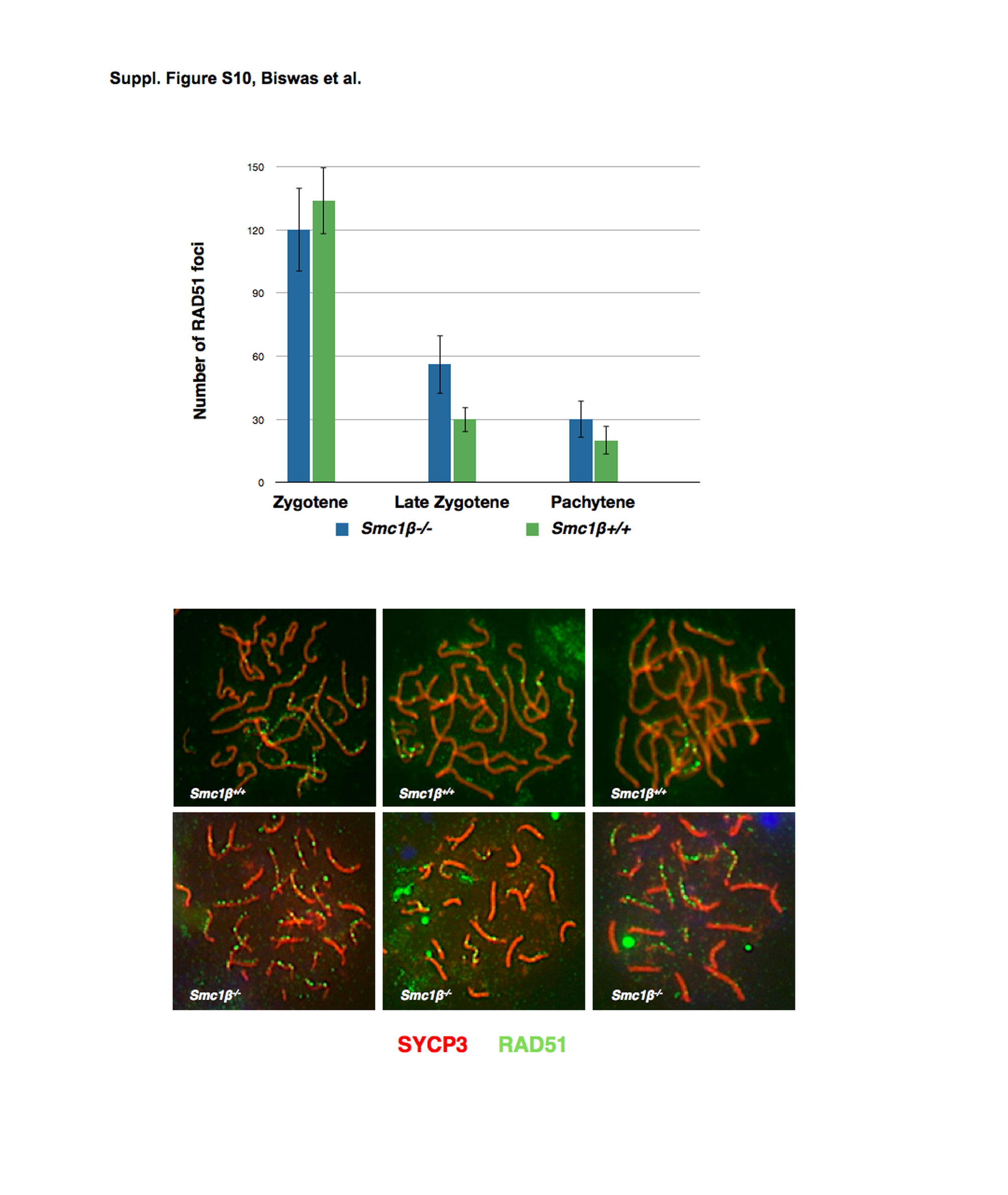

Supplement: Figure S10 — RAD51 foci in wt and Smc1β−/− spermatocytes. Spermatocyte spreads were stained with anti RAD51 and anti SYCP3. RAD51 foci were counted and the numbers of foci at different stages of meiosis is provided (n = 160). (TIFF) [file pgen.1003985.s010.tiff]
