# Supplementary material for: Meiotic Cohesin SMC1β Provides Prophase I Centromeric Cohesion and Is Required for Multiple Synapsis-Associated Functions
Source: PLoS Genet. 2013 Dec 26;9(12):e1003985. doi: 10.1371/journal.pgen.1003985 (PMC3873225; doi:10.1371/journal.pgen.1003985)
Supplement: Table S1 — Sequences of gene-specific primers for RT-PCR validation of microarray data. (DOCX) [file pgen.1003985.s011.docx]

**Suppl. Table 1, Biswas et al.**

Supplementary Table 1: Primer sequences for RT-PCR validation of microarray data.

| Gene symbol | Forward primer sequence | Reverse primer sequence |
| --- | --- | --- |
| *1700020N18Rik* | ACACAGAAACCCCACAGGTC | AATCCAGCAAGACCCACATC |
| *Aym1* | TCTACACACCCCTCCCTGTC | TTCCCTGAACTACCTGAGCAC |
| *4933426G20Rik* | ACAGGGCTGCTGGTACATCT | TCTCTATGGACTGGGCCAAC |
| *BU962292* | CCTGAACCTGAGTGGAGGAA | CATCCTGATGAGCGGGTAAT |
| *Odf4* | GGCTGGTGCTCATCCTGTAT | GACTCCTGGCTGTTCTCCTG |
| *Tmem30c* | AGGGAACTCACCCACTCTGA | GGGGAGGAGGAGCAAATAAA |
| *Lin7a* | ACATGGAGGCCTCAAAAGAG | AACGAGCCTCCATCTCTTCC |
| *1700006A11Rik* | ATGGAAAATGGCTTGCTGTC | AAAGCGTAAGCAGTCATGTGAG |
| *LOC625963* | CCCTGAGGGATAAGATTTACACTG | TTCATCAAGTACGCTAACCCAAT |
| *4930430A15Rik* | TGTATTTGGGTGGACTGCAT | TTGGGATGCTTGTCTAATGACTT |
| *Trdn* | CACCTGCTTGATTTCTGCAC | GGGGAGCTGAATGTTGTCAC |
| *9130204L05Rik* | TTATGCTCTGGGAACGACAG | TTCAAAGCCTCTTCAAAGCTG |
| *Pcdh8* | AAAGACAGCGGCAAAGGAG | AGCAGCGATCAGAATGACCT |
| *Foxg1* | GCCAGCAGCACTTTGAGTTA | GATCTCCCCGTTGACCAG |
| *Tcf1* | GCACACCCATGAAGACACAG | GGTTCCTACGCCCCTTCTTA |
| *1700080O16Rik* | ATGCGTGGAACCTGGTAGAC | CTGCCTCCAGGTCTCTTGTC |
| *Gm9* | GCCTCGGAATTGATGTGTCT | ACCATAGCCCTTGTGTTTGC |
| *Rhox3* | TGGGTGTGAATGAAGCCATA | ATAGTGGCACTGCAGGGTTC |
| *EG236892* | CGGTTTCCTGATGAGTGTCC | CCAGGTACTTCAGCTTCACCA |
| *1700010D01Rik* | TGCCATCAACCACAACATCT | ATCCTTCCCAGCGTTTTCTT |
| *EG667726* | TGAGCCCGAAGTAGAGGATG | AGGGAGTTCTCCAGACTGAGG |
| *Zfy1* | CAGATTGTGTTTCTGAAGCAGTCT | TCCTGACTCTGCATTCATGG |
| *Zfy2* | TCTGGAGCAGCAAGATGATG | TGCACACCTTGATAACTTCTGG |
| *Actb* | GGCTGTATTCCCCTCCATCG | CCAGTTGGTAACAATGCCATGT |
| *Gapdh* | GAGAAACCTGCCAAGTATGAC | GGAGTTGCTATGAAGTTGC |
