# Supplementary material for: Meiotic Cohesin SMC1β Provides Prophase I Centromeric Cohesion and Is Required for Multiple Synapsis-Associated Functions
Source: PLoS Genet. 2013 Dec 26;9(12):e1003985. doi: 10.1371/journal.pgen.1003985 (PMC3873225; doi:10.1371/journal.pgen.1003985)
Supplement: Table S2 — Up- and down-regulated genes in testes of 16 dpp Smc1β−/− mice. (DOCX) [file pgen.1003985.s012.docx]

**Suppl. Table 2, Biswas et al.**

Supplementary Table 2: Up- and down-regulated genes in testes of 16 dpp *Smc1β*^-/-^ mice.

| **Gene Number** | **Gene Symbol** | **FC** | **p-value** | **Chr** |
| --- | --- | --- | --- | --- |
|  |  |  |  |  |
| **Up-regulated** | | | | |
| 1 | Cypt6 | 19.9 | 2.10E-05 | X |
| 2 | Trdn | 13.5 | 4.68E-09 | 10 |
| 3 | 1700080O16Rik | 8.2 | 3.41E-05 | X |
| 4 | Trdn | 7.3 | 1.20E-05 | 10 |
| 5 | 4930432H15Rik | 6.6 | 3.19E-05 | X |
| 6 | C630007B19Rik | 6.1 | 3.91E-06 | 6 |
| 7 | 9130204L05Rik | 5.9 | 4.30E-05 | 3 |
| 8 | Gm9 | 5.6 | 6.01E-06 | X |
| 9 | Rhox3 | 5.5 | 2.80E-05 | X |
| 10 | EG236892 | 5.4 | 3.52E-06 | X |
| 11 | 1700010D01Rik | 5.4 | 1.63E-05 | X |
| 12 | Pcdh8 | 5.3 | 6.71E-06 | 14 |
| 13 | Evx1 | 5.2 | 3.78E-06 | 6 |
| 14 | Foxg1 | 5.1 | 6.38E-06 | 12 |
| 15 | EG667726 | 4.7 | 9.73E-06 | X |
| 16 | Tcf1 | 4.5 | 2.91E-05 | 10 |
| 17 | AI315523 | 4.4 | 1.66E-05 | 7 |
| 18 | Nkx2-5 | 4.3 | 6.40E-06 | 17 |
| 19 | Hpn | 4.1 | 6.37E-06 | 7 |
| 20 | Zfy2 | 4 | 3.52E-06 | Y |
| 21 | Cdh4 | 3.8 | 6.88E-07 | 2 |
| 22 | 4930534P07Rik | 3.8 | 3.24E-05 | X |
| 23 | Tcfap2e | 3.6 | 1.06E-07 | 4 |
| 24 | 3110039M20Rik | 3.5 | 2.68E-05 | 12 |
| 25 | Olfr114 | 3.4 | 1.09E-05 | 17 |
| 26 | Zfy1 | 3.3 | 7.75E-07 | Y |
| 27 | 3830403N18Rik | 3.3 | 8.70E-06 | X |
| 28 | Vax1 | 3.2 | 1.71E-05 | 19 |
| 29 | Rhox2 | 3 | 1.57E-06 | X |
| 30 | Hoxd1 | 3 | 1.93E-05 | 2 |
| 31 | 9130206I24Rik | 3 | 3.29E-05 | 4 |
| 32 | Tmem121 | 2.9 | 2.87E-05 | 12 |
| 33 | C730027E14Rik | 2.8 | 2.57E-05 | 11 |
| 34 | Tbx15 | 2.7 | 2.76E-05 | 3 |
| 35 | Gnas | 2.7 | 4.66E-05 | 2 |
| 36 | Nyx | 2.7 | 1.51E-05 | X |
| 37 | Areg | 2.6 | 2.19E-05 | 5 |
| 38 | Pet2 | 2.6 | 1.51E-05 | X |
| 39 | 3830417A13Rik | 2.6 | 9.07E-06 | X |
| 40 | Abcg4 | 2.6 | 2.57E-06 | 9 |
| 41 | 9130206I24Rik | 2.4 | 1.21E-05 | 4 |
| 42 | Elavl3 | 2.3 | 3.48E-06 | 9 |
| 43 | Rbmx | 2.3 | 3.81E-05 | X |
| 44 | Magea8 | 2.1 | 2.39E-05 | X |
| 45 | Magea3 | 2.1 | 3.52E-06 | X |
| 46 | Magea5 | 2.1 | 1.16E-05 | X |
| 47 | EG433637 | 2.1 | 4.22E-05 | 3 |
| 48 | Magea1 | 2.1 | 1.51E-05 | X |
|  |  |  |  |  |
| **Down-regulated** | | | | |
| 1 | 1700020N18Rik | 38.5 | 2.89E-06 | 1 |
| 2 | Aym1 | 37.5 | 1.72E-05 | 5 |
| 3 | 4933426G20Rik | 33.4 | 2.11E-05 | 15 |
| 4 | BU962292 | 27.5 | 4.71E-05 |  |
| 5 | Odf4 | 25.7 | 1.74E-05 | 11 |
| 6 | Tmem30c | 24.9 | 3.54E-05 | 16 |
| 7 | 1700006A11Rik | 24.7 | 1.94E-05 | 3 |
| 8 | LOC625963 | 24.6 | 2.45E-05 |  |
| 9 | 4930430A15Rik | 24.4 | 1.44E-05 | 2 |
| 10 | Hdhd1a | 24.3 | 1.88E-05 | 18 |
| 11 | 1700097M23Rik | 24.1 | 3.53E-05 | 6 |
| 12 | EG544888 | 23.5 | 3.44E-06 | 12 |
| 13 | 1700027A23Rik | 22.9 | 7.22E-06 | 3 |
| 14 | 4921525O09Rik | 22.4 | 1.98E-05 | 13 |
| 15 | BY706654 | 21.8 | 5.80E-06 |  |
| 16 | Armc3 | 21.2 | 2.26E-05 | 2 |
| 17 | 1700047I16Rik | 20.8 | 4.67E-05 | 13 |
| 18 | Gykl1 | 20.5 | 2.51E-05 | 18 |
| 19 | Lin7a | 20.3 | 8.18E-06 | 10 |
| 20 | 4931419H13Rik | 20 | 4.70E-05 | 3 |
| 21 | Cmtm2b | 19.8 | 2.26E-05 | 8 |
| 22 | Eps8l3 | 19 | 1.02E-06 | 3 |
| 23 | Sel1l2 | 19 | 4.01E-05 | 2 |
| 24 | Oplah | 19 | 9.31E-06 | 15 |
| 25 | Tmem30c | 18.8 | 7.14E-07 | 16 |
| 26 | 4933440M02Rik | 17.8 | 8.71E-06 | 7 |
| 27 | 1200003C05Rik | 17.4 | 2.75E-05 | 12 |
| 28 | Gm9880 | 17.3 | 3.60E-05 | 17 |
| 29 | NAP091803-1 | 17.1 | 3.87E-05 |  |
| 30 | 4930563E19Rik | 16.9 | 2.96E-05 | 1 |
| 31 | Tcp11 | 16.9 | 1.53E-05 | 17 |
| 32 | Spag16 | 16.6 | 1.53E-05 | 1 |
| 33 | Qrich2 | 16.6 | 1.12E-05 | 11 |
| 34 | Atp8b3 | 16.6 | 1.80E-05 | 10 |
| 35 | Wdr69 | 16.6 | 6.33E-06 | 1 |
| 36 | Gk2 | 16.4 | 2.95E-05 | 5 |
| 37 | Wdr63 | 16.3 | 7.49E-06 | 3 |
| 38 | Bpi | 16.1 | 1.89E-05 | 2 |
| 39 | D0H6S2654E | 16 | 2.14E-05 | 13 |
| 40 | Abca14 | 15.5 | 7.21E-06 | 7 |
| 41 | 1700030G11Rik | 14.6 | 3.54E-05 | 2 |
| 42 | Cetn1 | 14.6 | 4.38E-05 | 18 |
| 43 | 4933430H15Rik | 14.5 | 4.50E-05 | 3 |
| 44 | Pbp2 | 14.4 | 3.15E-05 | 6 |
| 45 | 4931408D14Rik | 14.4 | 2.94E-05 | 19 |
| 46 | 1700054H16Rik | 14 | 3.48E-05 | 11 |
| 47 | 4921537P18Rik | 13.9 | 1.62E-05 | 8 |
| 48 | Atb8b3 | 13.5 | 1.76E-05 | 10 |
| 49 | 5330417C22Rik | 13.4 | 1.69E-05 | 3 |
| 50 | Hjurp | 13.4 | 6.18E-06 | 1 |
| 51 | Ldhc | 13.2 | 3.70E-05 | 7 |
| 52 | 1700003M02Rik | 13.2 | 4.32E-06 | 4 |
| 53 | 4933409G03Rik | 13.1 | 2.68E-05 | 2 |
| 54 | Tekt1 | 13 | 1.15E-05 | 11 |
| 55 | Mospd4 | 12.9 | 1.83E-05 | 18 |
| 56 | 1700019N12Rik | 12.9 | 4.83E-05 | 19 |
| 57 | Wdr16 | 12.7 | 1.94E-05 | 11 |
| 58 | 4932414N04Rik | 12.7 | 4.67E-05 | 2 |
| 59 | Actl7b | 12.6 | 9.43E-06 | 4 |
| 60 | Cmtm1 | 12.5 | 2.62E-05 | 8 |
| 61 | Tdrd6 | 12.5 | 3.95E-05 | 17 |
| 62 | Sunc1 | 12.1 | 2.04E-05 | 11 |
| 63 | Lrriq1 | 12 | 1.38E-06 | 10 |
| 64 | 1110014J01Rik | 12 | 4.43E-08 | 15 |
| 65 | 4930538E20Rik | 11.9 | 1.82E-05 | 11 |
| 66 | Dnahc17 | 11.7 | 2.65E-05 | 11 |
| 67 | Ppp3r2 | 11.5 | 4.46E-05 | 4 |
| 68 | 4930465K10Rik | 11.5 | 4.84E-06 | 18 |
| 69 | 4932443J21Rik | 11.5 | 1.13E-05 | 9 |
| 70 | Tekt1 | 11.4 | 2.61E-06 | 11 |
| 71 | Ccdc7 | 11.3 | 1.78E-05 | 8 |
| 72 | BC050811 | 11.2 | 1.84E-05 | 3 |
| 73 | AK016580 | 11.2 | 2.13E-05 | 13 |
| 74 | Spag16 | 11.2 | 4.00E-06 | 1 |
| 75 | Ccdc19 | 11.1 | 2.48E-05 | 1 |
| 76 | 1700003M02Rik | 10.8 | 1.04E-06 | 4 |
| 77 | Ccdc42 | 10.7 | 1.55E-07 | 11 |
| 78 | Ttc18 | 10.6 | 4.24E-06 | 14 |
| 79 | Ccdc108 | 10.6 | 1.81E-05 | 1 |
| 80 | Speer2 | 10.2 | 1.64E-05 | 16 |
| 81 | Iqub | 10.2 | 4.68E-07 | 6 |
| 82 | Samd7 | 10.1 | 1.35E-06 | 3 |
| 83 | Dnahc6 | 10.1 | 6.42E-06 | 6 |
| 84 | Gtf2a1lf | 10 | 1.62E-06 | 17 |
| 85 | Rnf190 | 10 | 1.09E-05 | 11 |
| 86 | Mdh1b | 10 | 1.99E-05 | 1 |
| 87 | 4933412E24Rik | 10 | 1.24E-05 | 15 |
| 88 | 1700023E05Rik | 10 | 2.00E-05 | 5 |
| 89 | EG380907 | 9.9 | 2.23E-05 | 14 |
| 90 | 4930455B06Rik | 9.9 | 3.66E-06 | 15 |
| 91 | Mdh1b | 9.9 | 1.25E-05 | 1 |
| 92 | Slc2a5 | 9.8 | 8.40E-06 | 4 |
| 93 | 4931417E11Rik | 9.8 | 1.92E-05 | 6 |
| 94 | 4932701A20Rik | 9.8 | 2.39E-06 | 6 |
| 95 | LOC433772 | 9.7 | 4.28E-05 |  |
| 96 | Tex22 | 9.7 | 1.18E-05 | 12 |
| 97 | Btbd16 | 9.6 | 3.22E-06 | 7 |
| 98 | Rnf190 | 9.5 | 2.07E-05 | 11 |
| 99 | Spag6 | 9.5 | 2.90E-05 | 16 |
| 100 | 4930544G11Rik | 9.1 | 3.60E-05 | 6 |
| 101 | Lypd4 | 9.1 | 7.17E-06 | 7 |
| 102 | 4930519G04Rik | 9 | 4.87E-05 | 5 |
| 103 | ENSMUST00000053683 | 9 | 1.49E-05 |  |
| 104 | AK162044 | 9 | 4.24E-05 | 15 |
| 105 | 4930515G13Rik | 8.9 | 2.96E-05 | 17 |
| 106 | Dynlrb2 | 8.9 | 2.26E-06 | 8 |
| 107 | Slc22a16 | 8.9 | 1.26E-05 | 10 |
| 108 | Slc2a3 | 8.9 | 2.13E-05 | 6 |
| 109 | 4930553I04Rik | 8.9 | 8.26E-06 | 5 |
| 110 | Adam2 | 8.8 | 2.58E-05 | 14 |
| 111 | AK077196 | 8.8 | 1.03E-05 | 8 |
| 112 | Gm101 | 8.8 | 3.39E-05 | 1 |
| 113 | Ankrd60 | 8.8 | 1.75E-05 | 2 |
| 114 | Ms4a13 | 8.7 | 1.17E-05 | 19 |
| 115 | Armc4 | 8.6 | 4.04E-07 | 18 |
| 116 | 4931429I11Rik | 8.6 | 8.26E-06 | 9 |
| 117 | Rxfp2 | 8.6 | 1.43E-05 | 5 |
| 118 | Dynlrb2 | 8.5 | 3.73E-06 | 2 |
| 119 | Lrrc23 | 8.5 | 2.47E-05 | 6 |
| 120 | Armc4 | 8.4 | 1.92E-05 | 18 |
| 121 | 4933404M02Rik | 8.4 | 3.75E-06 | 2 |
| 122 | 4931429I11Rik | 8.3 | 2.35E-05 | 9 |
| 123 | 4933404M02Rik | 8.2 | 2.04E-06 | 2 |
| 124 | Dnali1 | 8 | 2.96E-05 | 4 |
| 125 | 4933415F23Rik | 8 | 4.88E-05 | 1 |
| 126 | NAP027583-1 | 8 | 1.58E-05 |  |
| 127 | Clec4g | 8 | 1.20E-05 | 8 |
| 128 | Dnajb13 | 7.9 | 9.52E-08 | 7 |
| 129 | Ccdc65 | 7.9 | 4.74E-05 | 15 |
| 130 | 4930418G15Rik | 7.8 | 2.23E-05 | 1 |
| 131 | BC024997 | 7.8 | 1.87E-05 | 11 |
| 132 | 1700021F07Rik | 7.8 | 1.67E-05 | 2 |
| 133 | Ankar | 7.8 | 4.61E-05 | 1 |
| 134 | 1700021F07Rik | 7.7 | 1.63E-05 | 2 |
| 135 | Spats1 | 7.7 | 2.95E-05 | 17 |
| 136 | Tmem45b | 7.7 | 4.10E-05 | 9 |
| 137 | B3gnt4 | 7.5 | 2.81E-05 | 5 |
| 138 | 1700010I14Rik | 7.5 | 4.65E-05 | 17 |
| 139 | Rshl1 | 7.5 | 3.39E-06 | 7 |
| 140 | Gm1060 | 7.4 | 7.10E-06 | 5 |
| 141 | Pp1r42 | 7.4 | 2.22E-05 | 1 |
| 142 | Insl6 | 7.4 | 2.74E-05 | 19 |
| 143 | Rshl1 | 7.4 | 5.21E-07 | 7 |
| 144 | Ropn1l | 7.3 | 4.55E-06 | 15 |
| 145 | Cdkl4 | 7.3 | 9.34E-07 | 17 |
| 146 | 4922502B01Rik | 7.2 | 3.91E-05 | 8 |
| 147 | Ccdc110 | 7.2 | 2.28E-06 | 8 |
| 148 | Iqcd | 7.2 | 3.66E-05 | 5 |
| 149 | EG545136 | 7.2 | 9.49E-06 | 15 |
| 150 | ENSMUST00000059746 | 7.2 | 1.20E-05 |  |
| 151 | Lrguk | 7.1 | 1.45E-06 | 6 |
| 152 | 1700017G19Rik | 7.1 | 3.48E-05 | 3 |
| 153 | Spats1 | 7.1 | 3.56E-05 | 17 |
| 154 | 4930404H11Rik | 7 | 3.45E-06 | 12 |
| 155 | Ankrd36 | 7 | 3.31E-05 | 11 |
| 156 | Acr | 6.9 | 8.51E-07 | 15 |
| 157 | 4930524O08Rik | 6.7 | 4.56E-05 | 9 |
| 158 | Daf2 | 6.6 | 4.71E-06 | 1 |
| 159 | Ypel1 | 6.6 | 7.48E-06 | 16 |
| 160 | Iqca | 6.6 | 1.76E-05 | 1 |
| 161 | 4933411G06Rik | 6.6 | 3.71E-05 | 10 |
| 162 | Cox8c | 6.5 | 2.51E-05 | 12 |
| 163 | Hydin | 6.5 | 4.49E-05 | 8 |
| 164 | Ropn1l | 6.4 | 6.05E-06 | 15 |
| 165 | 4930461L14Rik | 6.3 | 1.20E-05 | 18 |
| 166 | LOC677118 | 6.2 | 1.52E-05 |  |
| 167 | 4930444P10Rik | 6.2 | 3.35E-05 | 1 |
| 168 | D19Ertd652e | 6.2 | 5.39E-06 | 19 |
| 169 | Spdyb | 6.1 | 1.74E-05 | 5 |
| 170 | Gm70 | 6.1 | 4.27E-05 | 12 |
| 171 | 4930478A21Rik | 6.1 | 4.07E-06 | 15 |
| 172 | Ccdc113 | 6.1 | 2.62E-05 | 8 |
| 173 | Sept12 | 6.1 | 5.42E-06 | 16 |
| 174 | Cmtm2a | 6 | 2.83E-05 | 8 |
| 175 | B230215L15Rik | 6 | 3.10E-05 | 3 |
| 176 | Ttc25 | 6 | 3.73E-05 | 11 |
| 177 | Il4i1 | 5.9 | 3.96E-06 | 7 |
| 178 | 1700065I16Rik | 5.8 | 1.04E-05 | 15 |
| 179 | Papolb | 5.8 | 1.16E-05 | 5 |
| 180 | Lrrc50 | 5.8 | 5.76E-06 | 8 |
| 181 | 4921528I01Rik | 5.8 | 3.35E-05 | 18 |
| 182 | 4922502B01Rik | 5.8 | 3.88E-05 | 8 |
| 183 | Ccdc65 | 5.8 | 9.31E-06 | 15 |
| 184 | Cage1 | 5.7 | 2.97E-05 | 13 |
| 185 | NAP020355-001 | 5.7 | 4.99E-05 |  |
| 186 | Zswim2 | 5.6 | 1.87E-05 | 2 |
| 187 | Gstt2 | 5.5 | 6.62E-06 | 10 |
| 188 | 1700025K23Rik | 5.5 | 2.37E-05 | 10 |
| 189 | 1700040L02Rik | 5.4 | 1.09E-05 | 10 |
| 190 | ENSMUST00000063037 | 5.4 | 1.74E-05 |  |
| 191 | 1110020C03Rik | 5.2 | 1.07E-05 | 4 |
| 192 | 4933411G11Rik | 5.2 | 4.35E-05 | 5 |
| 193 | Zfa | 5.2 | 2.64E-05 | 10 |
| 194 | Pdcl2 | 5.2 | 2.12E-05 | 5 |
| 195 | Ak3 | 5.1 | 3.96E-05 | 19 |
| 196 | 4930589M24Rik | 5.1 | 8.51E-07 | 10 |
| 197 | Xrra1 | 5.1 | 1.66E-06 | 7 |
| 198 | Gramd1c | 5.1 | 4.78E-05 | 16 |
| 199 | Fam186b | 5.1 | 9.20E-06 | 15 |
| 200 | 2310015A05Rik | 5 | 4.05E-06 | 16 |
| 201 | Gm128 | 4.9 | 2.43E-05 | 3 |
| 202 | EG333669 | 4.9 | 3.29E-05 | 10 |
| 203 | AI464131 | 4.9 | 2.38E-05 | 4 |
| 204 | Slamf7 | 4.9 | 3.76E-05 | 1 |
| 205 | Tcte3 | 4.8 | 2.80E-05 | 17 |
| 206 | 1700028J19Rik | 4.8 | 1.10E-05 | 7 |
| 207 | Fank1 | 4.8 | 4.82E-05 | 7 |
| 208 | Ccdc60 | 4.7 | 4.40E-05 | 5 |
| 209 | 6430531B16Rik | 4.7 | 4.72E-05 | 7 |
| 210 | AK078072 | 4.7 | 1.99E-05 | 9 |
| 211 | Abca15 | 4.6 | 3.15E-05 | 7 |
| 212 | 1190002A17Rik | 4.6 | 7.39E-06 | 2 |
| 213 | Lrrc34 | 4.5 | 4.26E-05 | 3 |
| 214 | Ankrd5 | 4.5 | 1.26E-05 | 2 |
| 215 | Gm969 | 4.5 | 1.55E-05 | 19 |
| 216 | AK029617 | 4.5 | 1.66E-05 | 1 |
| 217 | OTTMUSG00000005148 | 4.4 | 2.86E-05 | 11 |
| 218 | Pde4dip | 4.4 | 2.98E-05 | 3 |
| 219 | Lrrc46 | 4.4 | 4.57E-05 | 11 |
| 220 | Ccdc105 | 4.3 | 5.61E-06 | 10 |
| 221 | D130043K22Rik | 4.3 | 1.92E-05 | 13 |
| 222 | Asrgl1 | 4.3 | 4.29E-05 | 19 |
| 223 | Lrrc6 | 4.2 | 6.06E-06 | 15 |
| 224 | Ccdc13 | 4.2 | 2.07E-05 | 9 |
| 225 | Kcnj9 | 4.1 | 2.45E-05 | 1 |
| 226 | Bspry | 4.1 | 6.11E-07 | 4 |
| 227 | Usp2 | 4 | 4.78E-05 | 9 |
| 228 | Rag1 | 4 | 3.16E-06 | 2 |
| 229 | Fhl4 | 4 | 3.11E-05 | 10 |
| 230 | D130043K22Rik | 4 | 8.91E-06 | 13 |
| 231 | Lrrc27 | 3.9 | 3.36E-05 | 7 |
| 232 | 4930544G21Rik | 3.9 | 7.17E-07 | 1 |
| 233 | Lrrc44 | 3.8 | 4.04E-05 | 3 |
| 234 | 2010015L04Rik | 3.8 | 3.67E-05 | 4 |
| 235 | LOC385319 | 3.8 | 4.62E-05 |  |
| 236 | Clgn | 3.8 | 3.08E-05 | 8 |
| 237 | Ccdc87 | 3.8 | 3.94E-05 | 19 |
| 238 | LOC627563 | 3.8 | 4.62E-05 |  |
| 239 | EG626359 | 3.7 | 3.23E-05 | 7 |
| 240 | Sgca | 3.6 | 1.54E-05 | 11 |
| 241 | Asb4 | 3.6 | 6.96E-06 | 6 |
| 242 | Lrrc50 | 3.6 | 1.23E-05 | 8 |
| 243 | Lrrc36 | 3.6 | 3.72E-05 | 8 |
| 244 | Wdr20b | 3.6 | 3.16E-05 | 12 |
| 245 | C330043M08Rik | 3.5 | 4.10E-05 | 6 |
| 246 | Tcam1 | 3.4 | 2.25E-05 | 11 |
| 247 | Phactr3 | 3.4 | 3.14E-06 | 2 |
| 248 | ENSMUSG00000046088 | 3.4 | 3.44E-06 | 17 |
| 249 | 4930503E14Rik | 3.3 | 1.89E-05 | 14 |
| 250 | Gm5134 | 3.3 | 6.98E-07 | 10 |
| 251 | Iqch | 3.3 | 3.51E-05 | 1 |
| 252 | Ypel1 | 3.2 | 4.32E-06 | 16 |
| 253 | Als2cr11 | 3.2 | 1.71E-05 | 1 |
| 254 | 4932431H17Rik | 3.2 | 8.58E-06 | 3 |
| 255 | Ribc2 | 3.2 | 4.43E-05 | 15 |
| 256 | Dyx1c1 | 3.2 | 6.86E-06 | 9 |
| 257 | AW456874 | 3.1 | 3.59E-05 | 13 |
| 258 | Rag1 | 3.1 | 4.01E-05 | 2 |
| 259 | Wdr66 | 3 | 2.16E-05 | 5 |
| 260 | Usp2 | 3 | 1.16E-05 | 9 |
| 261 | Lrrc29 | 3 | 2.58E-05 | 8 |
| 262 | 4930425N13Rik | 3 | 1.27E-05 | 9 |
| 263 | Ccdc74a | 3 | 3.25E-05 | 16 |
| 264 | Trim11 | 3 | 4.28E-05 | 11 |
| 265 | Bscl2 | 3 | 4.50E-05 | 19 |
| 266 | Glipr1l2 | 2.9 | 1.87E-05 | 10 |
| 267 | 4930521A18Rik | 2.9 | 2.58E-05 | 1 |
| 268 | Wdr17 | 2.9 | 2.36E-05 | 8 |
| 269 | Fhad1 | 2.9 | 4.15E-05 | 4 |
| 270 | Trim11 | 2.9 | 1.11E-05 | 11 |
| 271 | Tspyl4 | 2.9 | 6.08E-06 | 10 |
| 272 | Ccdc40 | 2.8 | 4.50E-05 | 11 |
| 273 | Abca16 | 2.8 | 4.59E-05 | 7 |
| 274 | Pex11c | 2.8 | 9.56E-06 | 8 |
| 275 | Aqp9 | 2.7 | 3.19E-06 | 4 |
| 276 | Tdrd1 | 2.7 | 1.38E-05 | 19 |
| 277 | Ccdc92 | 2.7 | 3.78E-05 | 5 |
| 278 | NAP061200-1 | 2.7 | 2.64E-05 | X |
| 279 | Ralgps1 | 2.7 | 2.98E-06 | 2 |
| 280 | Phf1 | 2.6 | 8.33E-06 | 17 |
| 281 | Gm166 | 2.6 | 2.26E-05 | 7 |
| 282 | Rage | 2.6 | 8.57E-06 | + |
| 283 | Ubxd5 | 2.6 | 4.90E-05 | 4 |
| 284 | Stk30 | 2.6 | 2.64E-05 | 12 |
| 285 | Gm9805 | 2.5 | 5.19E-06 | 17 |
| 286 | Csda | 2.4 | 3.08E-05 | 6 |
| 287 | Ift172 | 2.4 | 1.50E-05 | 5 |
| 288 | 1810046K07Rik | 2.4 | 3.23E-05 | 9 |
| 289 | BC043118 | 2.4 | 2.49E-05 | 16 |
| 290 | 6230410P16Rik | 2.3 | 6.35E-06 | 9 |
| 291 | 1700113I22Rik | 2.2 | 1.56E-05 | 11 |
| 292 | 9430057O19Rik | 2.2 | 7.51E-06 | 5 |
| 293 | Gstt3 | 2.2 | 3.23E-05 | 10 |
| 294 | Abhd8 | 2.2 | 9.57E-06 | 8 |
| 295 | Jmjd2d | 2.2 | 3.92E-05 | 9 |
| 296 | 4930503L19Rik | 2.1 | 3.88E-05 | 18 |
| 297 | Lrrc56 | 2 | 1.49E-05 | 7 |
| 298 | 4930544O15Rik | 2 | 3.37E-05 | 4 |

FC, fold change; Chr, chromosome.
